# Twenty-four-hour rhythmicity of circulating metabolites: effect of body mass and type 2 diabetes

**DOI:** 10.1096/fj.201700323R

**Published:** 2017-08-18

**Authors:** Cheryl M. Isherwood, Daan R. Van der Veen, Jonathan D. Johnston, Debra J. Skene

**Affiliations:** Chronobiology Section, Faculty of Health and Medical Sciences, University of Surrey, Guildford, United Kingdom

**Keywords:** metabolomics, human, obesity, biological rhythms, circadian clock

## Abstract

Metabolic profiling of individuals with type 2 diabetes mellitus (T2DM) has previously been limited to single-time-point samples, ignoring time-of-day variation. Here, we tested our hypothesis that body mass and T2DM affect daily rhythmicity and concentrations of circulating metabolites across a 24-h day in 3 age-matched, male groups—lean, overweight/obese (OW/OB), and OW/OB with T2DM—in controlled laboratory conditions, which were not confounded by large meals. By using targeted liquid chromatography/mass spectrometry metabolomics, we quantified 130 plasma metabolites every 2 h over 24 h, and we show that average metabolite concentrations were significantly altered by increased body mass (90 of 130) and T2DM (56 of 130). Thirty-eight percent of metabolites exhibited daily rhythms in at least 1 study group, and where a metabolite was rhythmic in >1 group, its peak time was comparable. The optimal time of day was assessed to provide discriminating biomarkers. This differed between metabolite classes and study groups—for example, phospholipids showed maximal difference at 5:00 AM (lean *vs.* OW/OB) and at 5:00 PM (OW/OB *vs.* T2DM). Metabolites that were identified with both robust 24-h rhythms and significant concentration differences between study groups emphasize the importance of controlling the time of day for diagnosis and biomarker discovery, offering a significant improvement over current single sampling.—Isherwood, C. M., Van der Veen, D. R., Johnston, J. D., Skene, D. J. Twenty-four-hour rhythmicity of circulating metabolites: effect of body mass and type 2 diabetes.

It is widely accepted that obesity is the main risk factor for type 2 diabetes mellitus (T2DM) (1). The progression from obesity to T2DM is largely a result of comorbidities, such as systemic inflammation and insulin resistance. Metabolic profiling by using targeted metabolomics, which enables the quantification of more than 100 low-MW intermediates of metabolism, is increasingly used to characterize (pre)diabetic phenotypes and has identified differences in metabolite profiles between those individuals who are obese and those with T2DM (2–5).

Recent work by our group and others has shown a 24-h variation in the human metabolome in healthy individuals, analyzed by using a range of analytical platforms (6–12), which has demonstrated that an estimated 15–20% of the metabolome is rhythmic in blood (6, 7). Transgenic mice that carry targeted genetic manipulation of circadian clock genes also exhibit a phenotype that involves defective metabolism, and associations between the circadian timing system and metabolic responses have been reported in humans (13). Reviews of these studies, including the higher incidence of obesity, T2DM, and related disorders in shift workers, have recently been published (14, 15).

Existing metabolomics studies in T2DM have been restricted to the analysis of single-time-point, mostly fasting, samples, which cannot characterize the effect of increased body mass and T2DM on rhythmic metabolites. Characterizing 24-h metabolite rhythms in T2DM compared with age- and body mass–matched controls may therefore provide novel insights into the etiology and progression of T2DM. Identification of the optimal time of day for blood sampling—when metabolite levels show the biggest difference between T2DM and controls—would also provide more discriminating diagnostic biomarkers, rather than taking a single morning fasting sample.

We thus assessed the effect of increased body mass [overweight/obese (OW/OB)] and T2DM on 24-h rhythms of circulating metabolites in men by using a quantitative targeted liquid chromatography/mass spectrometry (LC/MS) metabolomics approach. As T2DM is often accompanied by obesity, we set out to distinguish the effects of T2DM from those of increased body mass by incorporating both a lean and an OW/OB control group into the current study design.

## MATERIALS AND METHODS

All aspects of the study were conducted in accordance with the Declaration of Helsinki and conformed to international ethical standards. A favorable ethical opinion was obtained from the Surrey Research Ethics Committee and the University of Surrey Ethics Committee. Written, informed consent was obtained from all participants.

### Prelaboratory session

The screening process and study protocol have been described previously (16, 17). In brief, initial screening was conducted *via* a self-reported medical history questionnaire, and was confirmed by the participant’s general practitioner. Participants who were receiving medication that was known to influence the endogenous circadian timing system or who had a history of hypoglycemic episodes or hemoglobin A1c (HbA1c) > 8.5% (69 mM) were excluded. Additional clinical screening was performed to assess general health, sleep patterns, and diurnal preference (morningness/eveningness).

Effects of body mass index (BMI) and T2DM were assessed separately within the study by using 3 study groups of men: *1*) lean, *2*) OW/OB, and *3*) T2DM. Participants in the lean and OW/OB groups were matched for age and glucose homeostasis markers (HbA1c, fasting glucose and insulin), whereas participants in the OW/OB and T2DM groups were matched for age and BMI. Participant (*n* = 23) demographics for metabolomics analyses are shown in **Table 1**.

**TABLE 1.** Participant screening data of lean, OW/OB, and T2DM study groups

Variable	Lean, *n* = 8	OW/OB, *n* = 9	T2DM, *n* = 6
Age (yr)	53.6 ± 6.0	51.0 ± 7.7	57.3 ± 4.8
BMI (kg/m^2^)	23.2 ± 1.4***	29.8 ± 2.3^###^	32.2 ± 2.5
Waist circumference (cm)	88.9 ± 6.5***	105.1 ± 3.9^###^	112.8 ± 8.9
Glucose (mM)	4.2 ± 0.7***	4.9 ± 0.7**	6.7 ± 1.6
Insulin (pM)	28.1 ± 16.8**	39.9 ± 18.5**	110.5 ± 85.3
HbA1c (%)	5.4 ± 0.4***	5.3 ± 0.5***	6.9 ± 0.9
BP systolic (mmHg)	129.4 ± 9.7*	133.8 ± 11.1	146.8 ± 10.6
BP diastolic (mmHg)	84.0 ± 10.2	85.1 ± 10.7	88.8 ± 9.0

Data are given as means ± sd. BP, blood pressure. *P < 0.05, **P < 0.01, ***P < 0.001 *vs*. T2DM; ^###^*P* < 0.001 *vs*. lean (1 way ANOVA, Tukey *post hoc* test).

To minimize sleep debt and any effects of nutritional status or exercise, a strict prestudy protocol was prescribed the week before study entry at the Surrey Clinical Research Centre. Participants’ in-bed time was aligned to the sleep schedule of the laboratory session (10:30–6:30 AM), without deviation by more than 15 min. Within 90 min of waking, participants were asked to obtain at least 15 min of natural outdoor light to maximize circadian entrainment. Excessive exercise was also avoided. Compliance was verified by using continuously worn rest-activity monitors (Actiwatch-L; Cambridge Neurotechnology, Cambridge, United Kingdom). Alcohol and caffeine consumption was prohibited throughout the prestudy and laboratory session. For the first 4 d of the prestudy protocol, participants had to consume their meals within set time windows (breakfast 7:00–9:00 AM, lunch 12:00–2:00 PM, and dinner 5:30–7:30 PM) (16). During the last 3 d of the prestudy protocol, participants could only consume food that had been provided by the research team during these same time windows. Participants were given the same breakfast but could choose their other meals from a set selection of ready meals with similar macronutrient content. Total daily food energy (∼35% derived from fat) was 150% of the basal metabolic rate using the Schofield equation (18).

### Laboratory session

**Figure 1.**
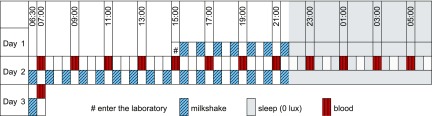
Study protocol. Participants (*n* = 23) entered the clinical laboratory at 3:00 PM on d 1 and lay semirecumbent throughout. The next day (d 2), during the light period from 6:30 AM to 10:30 PM (∼600 lux, in direction of gaze), participants were given hourly isocaloric milkshakes that commenced at 6:30 AM and water was available *ad libitum*. During the dark period (10:30 PM to 6:30 AM, 0 lux), they had the opportunity to sleep. Blood was drawn every 2 h from 7:00 AM on d 2 until 7:00 AM the next day (d 3).

During the laboratory session, light intensity, physical activity, and meals were strictly controlled, as illustrated in **Fig. 1**. Participants lay semirecumbent throughout. During lights on (6:30 AM–10:30 PM), light intensity was ∼600 lux in the direction of gaze. During the light period, small meals, which were composed of isocaloric Fortisip (Nutricia, Schiphol, The Netherlands) milkshakes, were given hourly from 3:30 PM (d 1), and water was available *ad libitum*. Individual food energy intake was set at 110% of the basal metabolic rate using the Schofield equation (18). During lights off (10:30 PM to 6:30 AM, 0 lux), participants had the opportunity to sleep.

Blood samples (*n* = 13 per participant) were drawn into anticoagulant (dipotassium-EDTA) tubes every 2 h from 7:00 AM on d 1 for 24 h, spun (3000 *g*, 10 min, 4°C) to isolate plasma, and stored at −80°C until analysis.

### Analysis of plasma metabolites

Samples for glucose and triglyceride (TAG) analyses were thawed overnight at 4°C before undergoing measurement in the ILab 650 Clinical Analyzer (Instrumentation Laboratory, North Risley, United Kingdom). The combined flow injection analysis and LC/MS targeted metabolomics analysis was performed by using the AbsoluteIDQ p180 kit (Biocrates Life Sciences AG, Innsbruck, Austria) and a Waters Xevo TQ-S mass spectrometer that was coupled to an Acquity UPLC system (Waters, Milford, MA, USA). Plasma samples (10 µl) were processed on four 96-well plates—each plate containing all time points from at least 1 participant from each group—and were measured within 1 mo of each other. The samples on each plate were randomly assigned to minimize any effect of assay drift.

Human plasma-based quality controls (QCs) with analytes added in 3 defined concentrations—low (QC1), medium (QC2), and high (QC3)—were provided with the kit. Medium quality control (QC2), repeated every 20 samples on each assay plate, was used to calculate the intra- and interplate coefficient of variation (CV%). Data were normalized between batches by using the results of QC2 repeats across the plate (*n* = 4) and between plates (*n* = 4) using METIDQ software (QC2 correction; Biocrates Life Sciences AG). Metabolites were excluded if the CV% of QC2 was >30% or if all 3 groups contained >25% of samples that were below the limit of detection, below the lower limit of quantification, or above the limit of quantification or blank out of range (*n* = 54 excluded) (19). Intraplate CV% of the remaining 130 quantified metabolites was 4.8 ± 0.3% (mean ± sem) and interplate CV% was 10.2 ± 0.5% (mean ± sem). Quantified metabolites were composed of 20 amino acids and 9 biogenic amines measured by LC/MS (7-point calibration using isotope-labeled internal standards) and 10 acylcarnitines, 13 lysophosphatidylcholines (lysoPCas), 30 diacylphosphatidylcholines (PCaas), 34 acyl-alkyl-phosphatidylcholines, 9 sphingomyelins (SMs), and 5 hydroxysphingomyelins measured by flow injection analysis (1-point calibration using isotope-labeled internal standards for acylcarnitines or unlabeled internal standards for lipids). Lipids were quantified by using nonphysiologic lipids as internal standards, 2 for PCs, 1 for lysoPCs, and 1 for sphingolipids. As a result of the unavailability of isotope-labeled lipid standards, quantification, but not precise identification, was possible (lipid signals measured with the Absolute*IDQ*p180 kit having a number of possible isobars and/or isomers).

### Data analysis

To assess 24-h metabolite rhythmicity, we performed cosinor analysis on mean *z*-score values—*z* score calculated across 24 h per individual (12)—for all participants. Peak time (acrophase), amplitude, and significance of a cosine fit (*P* < 0.05) were determined in each case (Matlab 2013b; MathWorks, Natick, MA, USA). Principal component analysis and orthogonal partial least squares discriminant analysis (OPLS-DA; SIMCA-P v.13.0 software; Umetrics, Malmo, Sweden) models (lean *vs.* OW/OB and OW/OB *vs.* T2DM) were used to identify metabolites with the most variance between study groups. Validation of the OPLS-DA models was provided by permutation analysis (999 permutations) and a CV ANOVA *P* value of the model. Differences in individual metabolite levels were analyzed in R (version 3.2.2; The R Foundation, Vienna, Austria) using the aov function in the statistics package (19). Linear models were fitted to the study group and time of day (13 time points, repeated measures per individual), with individual participants as covariates. Significant differences between study group, time of day, and their interaction were determined by using 2-way ANOVA. Significance values were corrected for multiple comparisons according to the Benjamini-Hochberg false discovery rate (FDR). Differences between metabolites were considered significant with an FDR <0.05. Missing values (*n* = 16; 5.4%) were left blank and were not taken into account for both the univariate and multivariate analyses. Missing values were not clustered in a particular sampling time (no time point had >4 missing samples) or study group (*n* = 7, 5, and 4 in lean, OW/OB, and T2DM groups, respectively). The time point at which most metabolites within each class had the maximum concentration difference between lean and OW/OB groups and between OW/OB and T2DM groups was calculated by using absolute values and illustrated in polar plots by using R. We performed quantitative enrichment analysis (lean *vs.* OW/OB and OW/OB *vs.* T2DM) by using MetaboAnalyst 3.0 (*www.metaboanalyst.ca*) (20).

## RESULTS

To assess the effect of increased body mass, we compared the OW/OB group with the lean group, both of which were matched for glucose homeostasis. Comparison of the OW/OB and T2DM groups enabled us to compare the effect of T2DM *per se* in weight-matched groups. Table 1 presents the screening demographic data for the 3 study groups. The T2DM group had significantly higher glucose, insulin, and HbA1c than both the OW/OB and lean groups (Table 1).

**Figure 2.**
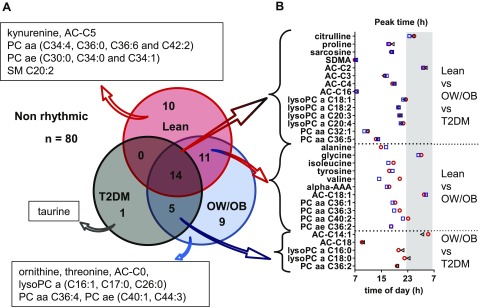
Metabolites with a significant cosine rhythm in the 3 study groups. *A*) Venn diagram showing the metabolites (name and number) with a significant cosine rhythm in the lean, OW/OB, and T2DM groups. *B*) Peak times of the metabolites with a significant cosine rhythm in more than 1 study group is also displayed. Peak time of day for each metabolite in the lean (red circle), OW/OB (blue square), and T2DM (black triangle) groups is shown. The dark condition (10:30 PM to 6:30 AM, 0 lux) is illustrated on the graph as a gray-shaded area, and time of day (hour) is displayed on the *x* axis.

**Figure 3.**
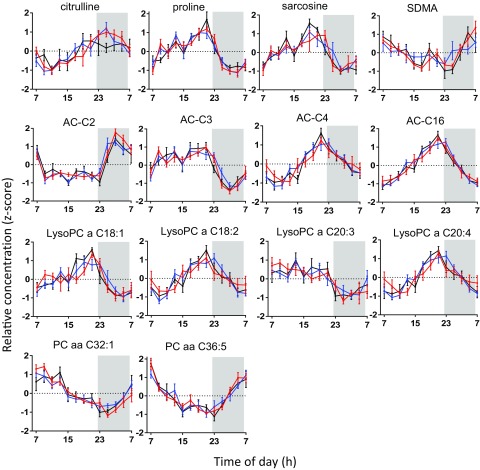
Metabolites with daily 24-h rhythms in all study groups. Metabolites with significant cosinor rhythms (*n* = 14) in the lean (red), OW/OB (blue), and T2DM (black) groups peak at a similar time of day, with no significant difference in relative amplitude. Relative concentration (*z* score) is displayed on the *y* axis, values are mean ± sem. The dark condition (22:30–06:30 h, 0 lux) is illustrated on the graph as a gray-shaded area, and time of day (h) is displayed on the *x* axis.

Assessment of daily rhythmicity, as determined by cosinor analysis of 24-h metabolite profiles, identified 50 (38%) of 130 metabolites with significant daily rhythms (*P* < 0.05) in at least one of the study groups (**Fig. 2**), 84% of which peaked during lights on. Fourteen (28%) of these 50 metabolites displayed 24-h daily rhythms in all 3 groups, namely, citrulline, proline, sarcosine, symmetrical dimethylarginine, acetylcarnitine, AC-C3 (propionylcarnitine), butyrylcarnitine, hexadecanoylcarnitine, 4× lysoPCa (C18:1, C18:2, C20:3, and C20:4), and 2× PCaa (C32:1 and C36:5). The 24-h profiles of these rhythmic metabolites are shown in **Fig. 3**. An additional 11 (22%) of 50 metabolites, namely, alanine, glycine, isoleucine, tyrosine, valine, α-aminoadipic acid (α-AAA), AC-C18:1 (octadecenoylcarnitine), PCaa (C36:1, C36:3, C40:2), and acyl-alkyl-phosphatidylcholine (C36:2) displayed daily rhythms in the lean and OW/OB groups, but not in the T2DM group, whereas 5 (10%) of 50 metabolites displayed daily rhythms in the OW/OB and T2DM groups, but not in the lean group, namely, tetradecenoylcarnitine and octadecanoylcarnitine, 2× lysoPCa (C16:0 and C18:0), and PCaa (C36:2). Also shown in Fig. 2, 20 (40%) of 50 metabolites displayed a daily rhythm in only 1 study group. Where a metabolite was rhythmic in >1 group [*n* = 30 (60%) of 50], its peak time was comparable between groups (within 2 h), with the exception of valine (6-h difference). Peak times of these metabolite rhythms are illustrated in Fig. 2 and are further detailed (peak time and amplitude) in Supplemental Table 1. There were no significant differences in the amplitude of metabolite rhythms between study groups. In addition to the panel of metabolites measured, plasma glucose concentrations displayed a significant cosine rhythm in the lean group only (*P* < 0.05), with a peak time of 15.02 h, and TAG displayed significant 24-h rhythms in the OW/OB and T2DM groups (*P* < 0.05), with peak times of 15.80 and 14.13 h, respectively.

**Figure 4.**
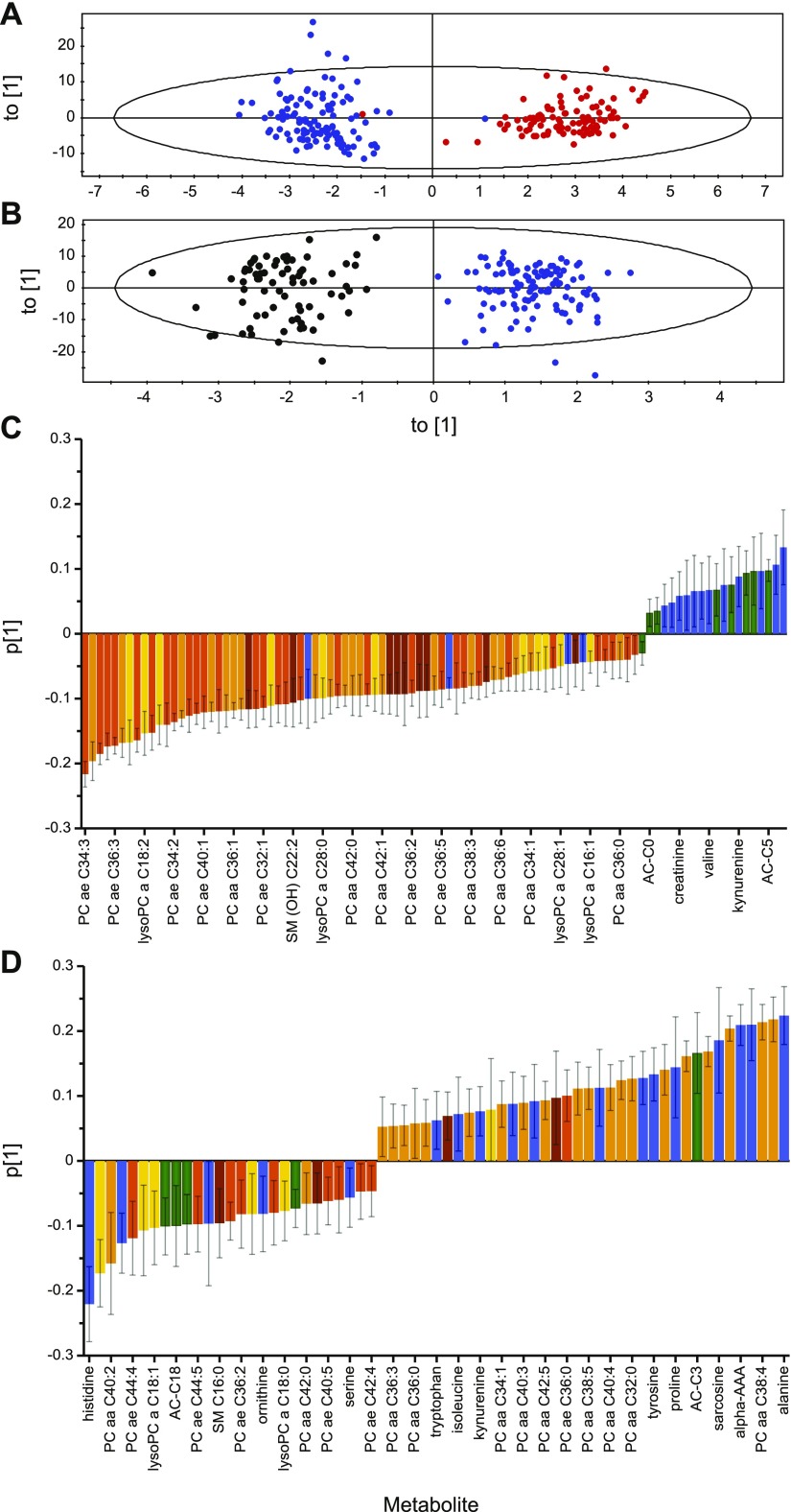
OPLS-DA plots separated according to study group. *A*) Score plot of lean (red) *vs.* OW/OB (blue) groups; *R*^2^*X* (cumulative), 0.636; *R*^2^*Y* (cumulative), 0.913; and *Q*^2^ (cumulative), 0.886 (validated by permutation analysis). *B*) Score plot of OW/OB (blue) *vs*. T2DM (black) groups; *R*^2^*X* (cumulative), 0.658; *R*^2^*Y* (cumulative), 0.913; and *Q*^2^ (cumulative), 0.879 (validated by permutation analysis). *C*) Loading plot of lean *vs.* OW/OB groups where negative *P* (loading) values represent metabolites with higher concentrations, and positive *P* (loading) values represent metabolites with lower concentrations in OW/OB compared with the lean group. Every fifth metabolite is labeled on the *x* axis. *D*) Loading plot of OW/OB *vs.* T2DM groups where negative *P* (loading) values represent metabolites with higher concentrations, and positive *P* (loading) values represent metabolites with lower concentrations in T2DM compared with the OW/OB group. Only metabolites whose error bars did not span the *x* axis have been plotted. Every second metabolite is labeled on the *x* axis. Metabolites are colored according to class; amino acids and biogenic amines (blue), acylcarnitines (green), PCaa (yellow), acyl-alkyl-phosphatidylcholine (PCae; light orange), lysoPC (dark orange), and sphingomyelin (brown). For a full list of all metabolites and their corresponding *P* (loading) values, see Supplemental Table 2.

A principal component analysis of all samples—130 metabolites, 13 time points, and 285 observations—was performed [*R*^2^*X* (cumulative), 0.877; *Q*^2^ (cumulative), 0.736; *n* = 16 components]. The OPLS-DA score plot of the lean and OW/OB groups in **Fig. 4*A*** demonstrates a clear separation of the 2 groups [*R*^2^*X* (cumulative), 0.636; *R*^2^*Y* (cumulative), 0.913; *Q*^2^ (cumulative), 0.886; *n* = 5 components, validated by permutation analysis; intercepts: *R*^2^ = 0.0, 0.187; and *Q*^2^ = 0.0, −0.435; and CV ANOVA *P* value lean *vs.* OW/OB, *P* = 2.12^−85^]. Variance within the model that was revealed in the loading plot (Fig. 4*B*) illustrates that the amino acids, biogenic amines, and acylcarnitines were mainly negatively correlated, whereas the phospholipids were mainly positively correlated within the model. The OPLS-DA score plot of the OW/OB and T2DM groups also demonstrates a clear separation of the groups [*R*^2^*X* (cumulative), 0.658; *R*^2^*Y* (cumulative), 0.913; *Q*^2^ (cumulative), 0.879; *n* = 5 components, validated by permutation analysis; intercepts: *R*^2^ = 0.0, 0.202; and *Q*^2^ = 0.0, −0.478; and CV ANOVA *P* value OW/OB *vs.* T2DM, *P* = 7.60^−75^; Fig. 4*C*]. In contrast, there was no obvious pattern in the variance between the OW/OB and T2DM groups according to metabolite class in the loadings plot (Fig. 4*D*). *P* (loading) values of all of the metabolites in both OPLS-DA models are shown in Supplemental Table 2.

We performed 2-way ANOVA (study group and time of day as factors) to assess the effect of increased body mass and T2DM on metabolite 24-h concentrations. Mean ± sem concentrations (micromolar) of the metabolites measured in the lean, OW/OB, and T2DM groups and of the metabolites with significant group differences (FDR < 0.05) between lean and OW/OB groups and OW/OB and T2DM groups are shown in Supplemental Table 3. Ninety metabolites (69%) were significantly different between the lean and OW/OB groups (FDR < 0.05), 70 (78%) of 90 had significantly lower concentrations in the OW/OB group, comprising mainly phospholipids [66 (94%) of 70], which confirmed the patterns that were observed in the OPLS-DA (Fig. 4). Quantitative enrichment analysis indicated that tyrosine metabolism (3-fold enrichment, *P* = 0.075), catecholamine biosynthesis (3-fold enrichment, *P* = 0.075), and phenylalanine and tyrosine metabolism (2-fold enrichment, *P* = 0.120) were the top 3 pathway-associated metabolite sets related to increased body mass. None of these pathways, however, was significantly enriched (*P* > 0.05).

**Figure 5.**
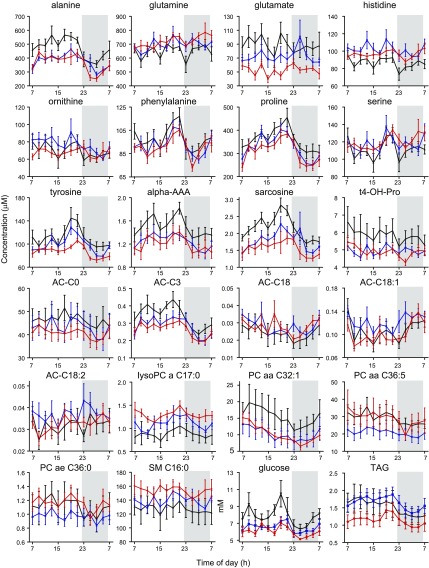
Twenty-four-hour profiles of metabolites, glucose, and TAG with significant concentration differences between OW/OB and T2DM study groups. Mean ± sem concentrations of all amino acids (*n* = 9), biogenic amines (*n* = 3), acylcarnitines (*n* = 5), and the most significantly different phospholipids in the lean (red), OW/OB (blue), and T2DM (black) study groups are shown. Concentration (micromolar for metabolites and millimolar for glucose and TAG) is shown on the *y* axis. The dark condition (10:30 PM to 6:30 AM, 0 lux) is illustrated on the graph as a gray-shaded area, and time of day (h) is displayed on the *x* axis.

When comparing T2DM and OW/OB groups, 56 (43%) of 130 metabolites demonstrated significant differences in concentration between the 2 groups (Supplemental Table 3). More than 60% of these metabolites [34 (61%) of 56] had significantly higher concentrations in T2DM, namely, amino acids (alanine, phenylalanine, glutamate, proline, and tyrosine), biogenic amines [α-AAA, sarcosine, *trans*-4-hydroxyproline (t4-OH-Pro)], acylcarnitines (AC-C0, AC-C3), 22× PC, and 2 sphingolipids (SM C20:2 and SM C26:1). Metabolites with significantly lower concentrations in T2DM [22 (39%) of 56] were glutamine, histidine, ornithine, serine, AC-C18 (octadecanoylcarnitine), AC-C18:1, octadecadienylcarnitine, 5× lysoPC, 8× PC, and 2 sphingolipids [SM C16:0 and SM(OH)C16:1]. The 24-h profiles of the metabolites with significant concentration differences between the T2DM group and OW/OB controls are shown in **Fig. 5**. The top 4 metabolite pathways that were associated with T2DM (between the OW/OB and T2DM study groups) were histidine metabolism (4-fold enrichment, *P* = 0.036), pyrimidine metabolism (2.5-fold enrichment, *P* = 0.123), purine metabolism (2.5-fold enrichment, *P* = 0.123), and glutamate metabolism (2.5-fold enrichment, *P* = 0.123). After FDR correction, the histidine metabolism pathway was not significantly enriched.

Figure 5 also shows the 24-h profiles of glucose and TAG. The 24-h average glucose levels were significantly higher (*P* < 0.001) in the T2DM group (mean ± sem, 7.74 ± 0.32 mM) compared with the OW/OB group (6.53 ± 0.15 mM) and lean controls (6.08 ± 0.6 mM). The 24-h average TAG concentrations were significantly lower (*P* < 0.01) in the T2DM group (1.43 ± 0.05 mM) compared with the OW/OB group (1.72 ± 0.23 mM), and were significantly higher (*P* < 0.001) in the OW/OB group compared with lean controls (1.15 ± 0.04 mM).

Six metabolites—proline, sarcosine, AC-C3, lysoPCa (C18:1 and C18:2), and PCaa (C36:5)—were affected by body mass, T2DM, and time of day (*i.e.*, had robust daily rhythms in every study group and significant concentration differences between study groups and their controls). These were the only metabolites with significant differences in concentration between study groups that also exhibited significant 24-h cosine rhythms in most of the participants in all study groups. Proline, sarcosine, and AC-C3 had significantly higher concentrations in the OW/OB group compared with the lean group that were significantly higher still in the T2DM group compared with the OW/OB group. Lysophosphatidylcholines (C18:1 and C18:2) had significantly lower concentrations in the OW/OB group compared with the lean group that were significantly lower still in the T2DM group compared with the OW/OB group. Diacylphosphatidylcholine (PCaa C36:5) did not follow this pattern, its concentrations being significantly lower in the OW/OB group compared with the lean group and significantly higher in the T2DM group compared with the OW/OB group.

**Figure 6.**
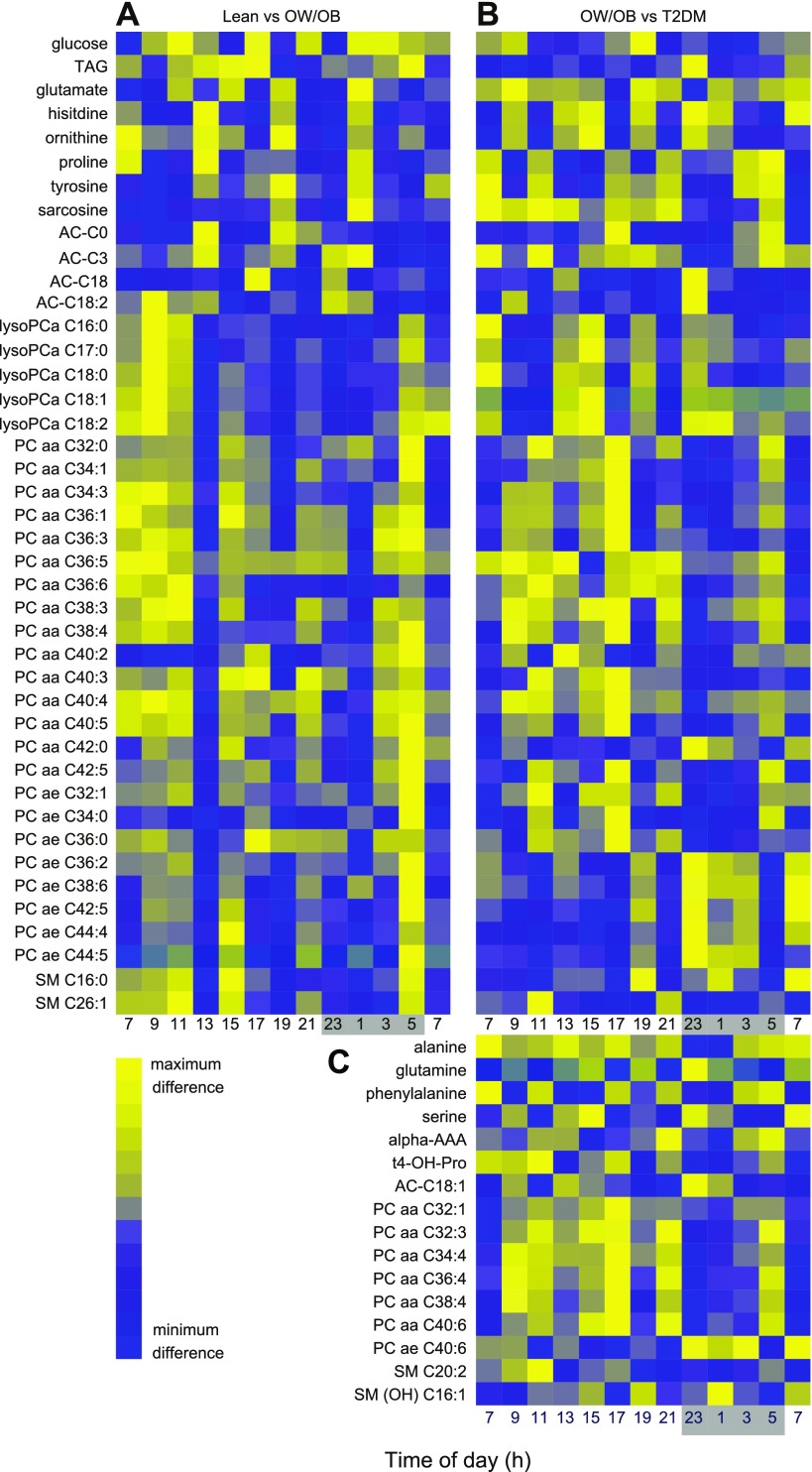
Heatmaps showing optimal time of day for maximizing sampling differences between study groups. *A*) Time points of maximum (yellow) or minimum (blue) concentration difference between OW/OB and lean groups across 24 h. *B*, *C*) Time points of maximum (yellow) or minimum (blue) concentration difference between T2DM and OW/OB groups across 24 h. The dark condition (10:30 PM to 6:30 AM, 0 lux) is illustrated on the *x* axis as a gray-shaded area.

In addition to the effect of body mass and T2DM, more than one half of the metabolites [80 (61%) of 130] exhibited a significant variation with time of day (ANOVA, FDR < 0.05; Supplemental Table 3). No metabolites showed a significant time of day × group interaction, with the exception of lysoPCa 16:1 (lean *vs.* OW/OB group). We calculated the time point that best distinguished concentration differences between study groups. Metabolites that had significant concentration differences in both the OW/OB group compared with lean controls and the T2DM group compared with OW/OB controls—comprising one third of the metabolites [*n* = 40 (31%) of 130]—were used to determine the optimal sampling time. Concentration differences for these metabolites at each time point across 24 h are plotted as heatmaps (**Fig. 6**) and show the optimal time of day for sampling to maximize concentration differences between groups. For each metabolite, the time point at which its concentration differed the most between study groups is included in Supplemental Table 3. We also calculated the time point at which most metabolites within each class had the maximum concentration difference between study groups. The polar plot of these data that compares the lean and OW/OB groups (Supplemental Fig. 1*A*) illustrates that 30% of the amino acids and biogenic amines classes (combined) had their maximum differences in concentration at 1:00 AM. In contrast, 30% of acylcarnitines had their maximum differences at 1:00 PM and 45% of the phospholipids had their maximum differences at 5:00 AM. Comparing the OW/OB and T2DM groups (Supplemental Fig. 1*B*), 20% of the amino acids and biogenic amines (combined) had their maximum differences in concentration at 1:00 and 7:00 AM. In contrast, 40% of acylcarnitines had their maximum differences at 23:00 h, and 30% of phospholipids had their maximum differences at 5:00 PM, 10 and 12 h different from the lean and OW/OB groups, respectively. The optimum time of day to detect differences between study groups thus differed according to metabolite class and the study groups being compared.

## DISCUSSION

This study, to our knowledge, is the first to independently map the effects of increased body mass and the combined effects of obesity and T2DM on 24-h plasma metabolite rhythms in men. Rhythmic metabolites were observed in all study groups (lean, *n* = 35; OW/OB, *n* = 39; T2DM, *n* = 20), 84% of which peaked during lights on. Metabolites that demonstrated significant daily rhythms are involved in the biochemical pathways that underlie T2DM and have been proposed as biomarkers (*e.g.*, branched chain amino acids, short chain acylcarnitines, α-AAA) (21–25). The robust time-of-day variation that was observed in the metabolite profiles highlights that time of day should be noted and controlled for clinical sampling, biomarker discovery, and interpretation of T2DM metabolomics studies.

Metabolites that exhibit significant cosine rhythms in more than 1 study group (*n* = 30) peaked at approximately the same time of day, thus suggesting that the timing of metabolite rhythms is not significantly affected by increased body mass or T2DM. This lack of change in the timing of metabolite rhythms is in agreement with our earlier work that showed no change in the timing of the plasma melatonin and leptin rhythms in these study groups (17). Similarly, there was no significant difference in the amplitude of the rhythmic metabolites between study groups. This finding contrasts with reports of attenuated gene expression circadian rhythmicity in adipose tissue in mouse models of obesity and T2DM (26) and in leukocytes of patients with T2DM (27). The disparity between the attenuated rhythms observed in gene expression and the retention of plasma metabolite rhythms in obesity and T2DM likely reflects the homeostatic mechanisms and multiple regulatory pathways that are present in the circulation. The comparable robust metabolite rhythms that were observed in both the OW/OB and T2DM groups indicate that no general rule of reduced amplitude from lean to obese to T2DM can be made.

Daily rhythms in plasma metabolites have previously been characterized in lean, young male study participants by using the same targeted LC/MS-based metabolomics approach (12). Of the 171 metabolites measured in that study, 109 (64%) exhibited a diurnal rhythm compared with only 27% rhythmic metabolites in the lean group in the present study. Although the participants in both studies had similar sleep-wake times, participants in the study by Davies *et al.* (12) had regular meals—breakfast, lunch, dinner, evening snack—rather than the hourly isocaloric meals in the present study, which resulted in set, imposed postprandial changes in circulating metabolites. In the present study, choosing to administer hourly isocaloric meals during the light period provided the opportunity to test postabsorptive metabolism while removing the imposed effects of large meals on metabolite profiles. Despite these differences in study protocols, significant 24-h cosine rhythms were observed in some of the same metabolites of the study by Davies *et al.* (12) and the lean group of the present study, namely, isoleucine, proline, valine, kynurenine, symmetrical dimethylarginine, sarcosine, α-AAA, acetylcarnitine, butyrylcarnitine, hexadecanoylcarnitine, AC-C18:1, lysoPCa C18:2, PCaa C40:2, PCaa C42:2, and SM C20:2. Both glucose and TAG levels peaked in the afternoon, which is in agreement with earlier studies under similar, regular, small meal feeding conditions (28), and are most likely a result of insulin resistance in the afternoon. Whereas nutritional status undoubtedly affects circulating metabolite levels, there is also evidence to suggest that some of these metabolites are under circadian clock control—rhythmic expression being present in laboratory constant routine conditions that are optimized to reveal endogenous circadian rhythms (8, 9, 11, 28).

Previous studies that have compared the metabolic profiles of individuals who went on to develop T2DM with those that did not have helped to uncover novel T2DM biomarkers (21–25). Although not all studies identified the same metabolites, a T2DM metabolic phenotype may include higher concentrations of alanine, glutamate, phenylalanine, tyrosine, α-AAA, t4-OH-Pro, and branched-chain amino acids (leucine, isoleucine, and valine), as well as lower concentrations of glycine, serine, and glutamine. Of note, in the present study, significantly higher alanine, phenylalanine, α-AAA, and t4-OH-Pro levels, as well as lower glutamine and serine levels were only observed in T2DM compared with the OW/OB group and, therefore, may arise independently of increased body mass. Individuals with α-AAA concentrations in the top quartile show the strongest association with diabetes risk, high α-AAA levels being associated with insulin resistance and impaired β-cell function (24). Elevated α-AAA levels are associated with low concentrations of glutathione, likely as a result of inflammation and oxidative stress, which are common comorbidities of T2DM (21) that are consistent with the raised α-AAA levels in our T2DM group. Elevated daytime α-AAA concentrations also resulted in loss of the α-AAA rhythm in the T2DM group. Alterations in the shape of the 24-h profiles, which resulted in loss of the cosine rhythm, in some key amino acids that are involved in T2DM—namely, alanine, glycine, isoleucine, tyrosine, and valine—suggest defective 24-h processing in T2DM that requires additional study.

Metabolites that demonstrated significant changes in OW/OB compared to the lean group, and in T2DM compared to the OW/OB group could be on a continuum from lean to obesity to T2DM. For example, in the current study, significantly higher short-chain acylcarnitine (AC-C0 and AC-C3) levels were observed in the OW/OB and T2DM groups. Similar findings have also been reported previously (29), with higher acylcarnitine concentrations possibly resulting from excessive nonesterified fatty acid (NEFA) availability, reduced NEFA oxidation, or both, as malonyl-CoA inhibition of carnitine palmitoyltransferase 1 slows the transfer of NEFA into the mitochondria (29).

T2DM had no consistent effect on phospholipids, with both higher (*n* = 22) and lower levels (*n* = 13) in the T2DM group compared with the OW/OB control group. The effect of T2DM on specific phospholipid species varies between metabolomics studies, so much so that a meta-analysis could not be performed on the lipid class as a result of high interstudy inconsistency (25). Plasma lysoPCs were negatively associated with BMI and plasma insulin concentrations, but had no relationship with glucose and the homeostatic model assessment-insulin resistance, which suggests that adiposity underlies the observed changes rather than insulin resistance or T2DM (5); however, in the current study, lysoPC responses seem to be more consistent—lysoPCs with significant group differences had lower levels in the OW/OB group that were lower still in T2DM, which suggests that they are affected by both weight and T2DM. Rhythmic lysoPCs also displayed robust rhythms, with the exception of C26:1, that all peaked in the evening.

We performed quantitative enrichment analysis; however, no pathways were identified as being significantly over-represented after FDR correction. Several metabolites that exhibited robust daily rhythms in the majority of participants in all study groups and that also showed a significant progressive change in concentration from lean to OW/OB to T2DM groups were identified. These metabolites, namely, proline, sarcosine, AC-C3, and lysoPCa (C18:1 and C18:2), may play a role or act as biomarkers in the progression from a healthy weight to obese with T2DM. These metabolites are worthy of future study to identify underlying mechanisms and to increase our understanding of the interaction between T2DM and the circadian timing system.

The time series sampling also afforded the opportunity to examine the optimum sampling time at which to best distinguish differences between study groups. For each metabolite, an optimum time of collection was determined to provide better discriminating biomarkers. The rhythmic nature of most metabolites meant that the best sampling time differed depending on the metabolite, metabolite class, and the study groups being compared. We observed some large differences (10–12 h) between study groups, for example, acylcarnitines showed maximal difference at 1:00 (lean *vs.* OW/OB) and at 11:00 PM (OW/OB *vs.* T2DM), and phospholipids showed maximal difference at 5:00 AM (lean *vs.* OW/OB) and at 5:00 PM (OW/OB *vs*. T2DM). In contrast, the optimum sampling time for amino acids and biogenic amines was 01:00 h, irrespective of the study groups being compared. Despite being unable to generate any general rules for the optimum sampling of metabolites, it is interesting to note that none of the metabolite classes exhibited its maximum concentration differences between 7:00 AM and 11:00 PM, when samples are typically drawn for diagnostic and research purposes. These findings highlight the importance of controlling the time of day and highlight the value of metabolic profiling across 24 h.

## CONCLUSIONS

To our knowledge, this is the first time a 24-h time series of plasma metabolites has been simultaneously assessed in T2DM compared with an age- and weight-matched control group during a controlled daily routine. It is clear that many aspects of the altered metabolome in T2DM exhibit 24-h diurnal variation, which has previously been overlooked in studies that used only single samples that were taken at a poorly defined time point. Our results reveal that the effect of body mass and T2DM on the rhythmic metabolome vary in a metabolite-specific manner. Furthermore, our findings highlight how the time of day regulates metabolite differences between lean, OW/OB, and T2DM phenotypes. These data thus increase our understanding of the interactions between circadian and metabolic physiology and will inform the experimental design of future studies.

## Supplementary Material

Supplemental Data

## Abbreviations

α-AAA: α-aminoadipic acid
AC-C18:1: octadecenoylcarnitine
AC-C3: propionylcarnitine
BMI: body mass index
CV: coefficient of variation
FDR: false discovery rate
HbA1c: hemoglobin A1c
LC/MS: liquid chromatography/mass spectrometry
lysoPCa: lysophosphatidylcholine
NEFA: nonesterified fatty acid
OPLS-DA: orthogonal partial least squares discriminant analysis
OW/OB: overweight/obese
PCaa: diacylphosphatidylcholine
QC: quality control
SM: sphingomyelin
T2DM: type 2 diabetes mellitus
t4-OH-Pro: *trans*-4-hydroxyproline
TAG: triglyceride
