# Supplementary material for: Twenty-four-hour rhythmicity of circulating metabolites: effect of body mass and type 2 diabetes
Source: FASEB J. 2017 Aug 18;31(12):5557–67. doi: 10.1096/fj.201700323R (PMC5690388; doi:10.1096/fj.201700323R)
Supplement: Supplemental Data [file supp_31_12_5557__index.html]

Twenty-four-hour rhythmicity of circulating metabolites: effect of body mass and type 2 diabetes — Twenty-four-hour rhythmicity of circulating metabolites: effect of body mass and type 2 diabetes — Supplemental Data 

# Twenty-four-hour rhythmicity of circulating metabolites: effect of body mass and type 2 diabetes

## Supplemental Data

- Supplemental Data
- Supplemental Data
- Supplemental Data
- Supplemental Data
