# Supplementary material for: Twenty-four-hour rhythmicity of circulating metabolites: effect of body mass and type 2 diabetes
Source: FASEB J. 2017 Aug 18;31(12):5557–67. doi: 10.1096/fj.201700323R (PMC5690388; doi:10.1096/fj.201700323R)
Supplement: Supplemental Data [file supp_fj.201700323R_Supplemental_Figure.pdf]

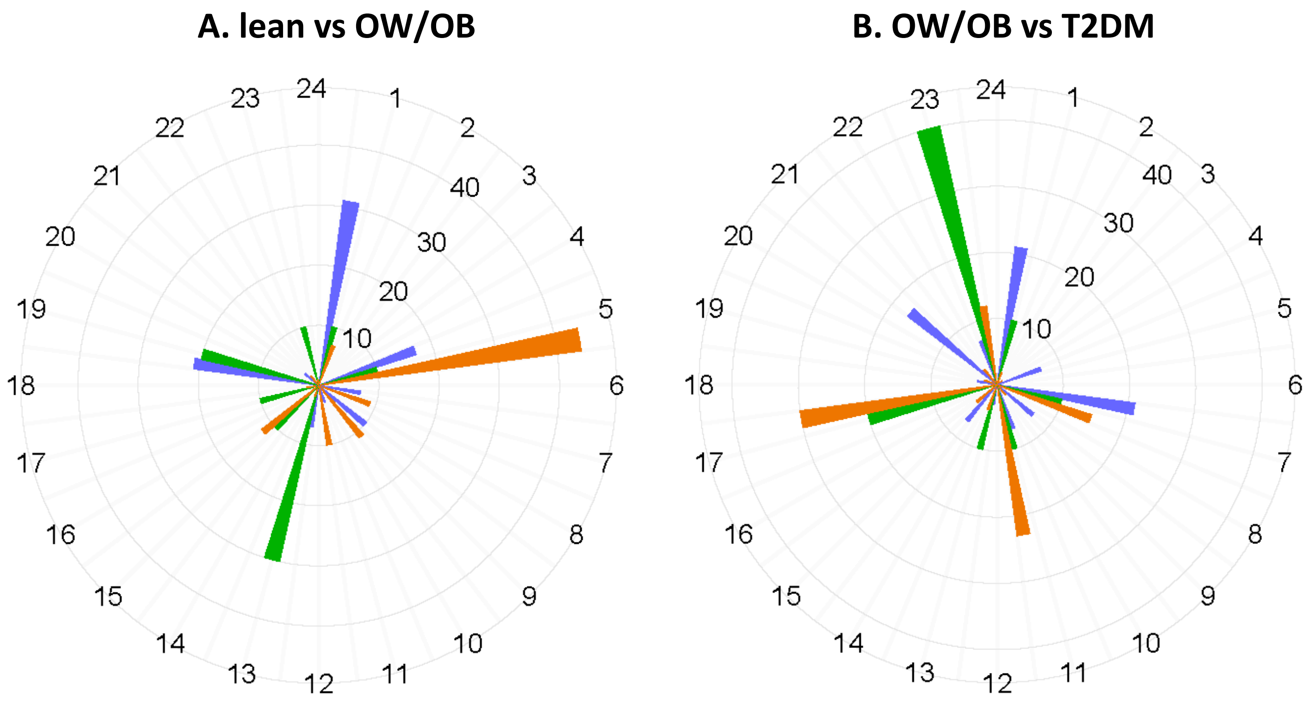

Figure S1 Polar plots showing time of day at which most metabolites within a class have the largest concentration difference between A. the lean and OW/OB groups and B. the OW/OB and T2DM groups. Time of day (h) is shown on the circumference and the percentage of metabolites within each class showing the largest difference between the study groups is shown on the radius of each plot. Metabolites classes are colored; amino acids and biogenic amines combined (blue), acylcarnitines (green), phospholipids (orange). In A. comparing the lean and OW/OB groups, the optimum time of day to detect differences in over 30% of the amino acids and biogenic amines (combined) was at 01:00 h, whereas 30% of the acylcarnitines and 45% of the phospholipids had their maximum differences at 13:00 h and 05:00 h, respectively. In B. comparing OW/OB and T2DM, the optimum time of day to detect differences in 20% of the amino acids and biogenic amines (combined) was 01:00 h and 07:00 h, 23:00 h for acylcarnitines (40%) and 17:00 h for phospholipids (30%).
