# Supplementary material for: Twenty-four-hour rhythmicity of circulating metabolites: effect of body mass and type 2 diabetes
Source: FASEB J. 2017 Aug 18;31(12):5557–67. doi: 10.1096/fj.201700323R (PMC5690388; doi:10.1096/fj.201700323R)
Supplement: Supplemental Data [file supp_fj.201700323R_Supplemental_Table1.docx]

**Table S1.** Metabolites with a significant 24 h cosinor rhythm (peak time and relative amplitude (amp)) in the lean, OW/OB and T2DM groups

|  | lean (n = 8) | | |  |  | OW/OB (n = 9) | | | |  |  | T2DM (n = 6) | | |  |
| --- | --- | --- | --- | --- | --- | --- | --- | --- | --- | --- | --- | --- | --- | --- | --- |
| Metabolite | p value | peak time | amp | n of 8 |  |  | peak time | amp | n of 9 | |  | p value | peak time | amp | n of 6 |
| citrulline | 2.75E-06 | 0.92 | 0.94 | 4 |  | 1.18E-04 | 23.53 | 0.87 | 3 | |  | 4.68E-03 | 0.46 | 0.64 | 3 |
| AC-C2 | 5.24E-03 | 3.99 | 0.97 | 5 |  | 2.90E-03 | 3.65 | 0.94 | 7 | |  | 5.94E-03 | 4.37 | 0.91 | 4 |
| **SDMA** | **2.87E-03** | **6.91** | **0.72** | **2** |  | **2.73E-03** | **7.24** | **0.38** | **2** | |  | **4.06E-02** | **7.24** | **0.72** | **2** |
| **AC-C16** | **1.03E-03** | **7.24** | **0.93** | **5** |  | **1.94E-04** | **7.48** | **0.83** | **5** | |  | **1.22E-03** | **7.06** | **0.97** | **5** |
| **PC aa C32:1** | **3.04E-03** | **10.82** | **0.94** | **6** |  | **6.60E-04** | **9.81** | **0.87** | **5** | |  | **2.90E-03** | **10.64** | **0.86** | **6** |
| **PC aa C36:5** | **1.74E-02** | **13.34** | **0.69** | **2** |  | **4.63E-03** | **13.94** | **0.61** | **2** | |  | **4.10E-02** | **13.98** | **0.64** | **4** |
| **AC-C3** | **1.26E-02** | **15.98** | **0.83** | **5** |  | **2.53E-03** | **16.56** | **0.83** | **5** | |  | **6.48E-03** | **15.77** | **0.85** | **3** |
| **proline** | **3.09E-03** | **17.40** | **0.91** | **5** |  | **7.96E-04** | **17.08** | **0.87** | **6** | |  | **1.17E-02** | **18.33** | **0.82** | **4** |
| **sarcosine** | **4.64E-03** | **17.94** | **0.71** | **4** |  | **3.61E-04** | **18.21** | **0.73** | **4** | |  | **7.38E-03** | **17.94** | **0.90** | **6** |
| **AC-C4** | **4.59E-02** | **18.35** | **0.57** | **2** |  | **5.31E-04** | **18.98** | **0.73** | **7** | |  | **9.28E-03** | **18.69** | **0.84** | **5** |
| **lysoPC a C18:2** | **1.32E-06** | **20.20** | **1.01** | **5** |  | **2.14E-07** | **20.09** | **1.14** | **9** | |  | **2.22E-05** | **20.23** | **1.12** | **6** |
| **lysoPC a C20:3** | **8.96E-04** | **20.52** | **0.64** | **5** |  | **1.39E-05** | **20.41** | **0.97** | **8** | |  | **7.21E-04** | **20.78** | **0.92** | **2** |
| **lysoPC a C20:4** | **1.04E-03** | **21.65** | **0.68** | **5** |  | **8.79E-08** | **20.85** | **1.04** | **9** | |  | **3.53E-04** | **21.03** | **0.96** | **5** |
| **lysoPC a C18:1** | 1.81E-04 | 22.42 | 0.76 | 5 |  | **4.35E-07** | **21.60** | **1.00** | **8** | |  | **2.53E-04** | **21.98** | **1.04** | **5** |
| glycine | 1.97E-02 | 3.13 | 0.62 | 3 |  | 2.00E-02 | 2.27 | 0.54 | 3 | |  |  |  |  |  |
| AC-C18:1 | 2.30E-02 | 3.99 | 0.68 | 3 |  | 2.78E-02 | 4.50 | 0.47 | 2 | |  |  |  |  |  |
| **alanine** | **1.67E-02** | **14.87** | **0.84** | **3** |  | **1.28E-03** | **16.37** | **0.68** | **4** | |  |  |  |  |  |
| **tyrosine** | **3.94E-02** | **17.38** | **0.66** | **3** |  | **2.87E-03** | **18.36** | **0.78** | **7** | |  |  |  |  |  |
| **alpha-AAA** | **1.18E-02** | **17.38** | **0.65** | **2** |  | **1.93E-03** | **16.65** | **0.74** | **5** | |  |  |  |  |  |
| **PC ae C36:2** | **4.02E-02** | **17.94** | **0.38** | **2** |  | **1.38E-02** | **17.90** | **0.49** | **1** | |  |  |  |  |  |
| **isoleucine** | **9.15E-03** | **18.64** | **0.61** | **2** |  | **2.48E-02** | **17.08** | **0.57** | **1** | |  |  |  |  |  |
| **PC aa C36:1** | **1.23E-02** | **19.17** | **0.45** | **2** |  | **5.81E-03** | **18.39** | **0.49** | **0** | |  |  |  |  |  |
| **PC aa C36:3** | **3.59E-02** | **20.55** | **0.41** | **4** |  | **4.45E-03** | **18.84** | **0.52** | **3** | |  |  |  |  |  |
| **valine** | **6.44E-03** | **20.57** | **0.56** | **1** |  | **3.32E-02** | **14.28** | **0.38** | **1** | |  |  |  |  |  |
| **PC aa C40:2** | 2.20E-02 | 23.12 | 0.62 | 4 |  | **1.64E-02** | **21.52** | **0.41** | **1** | |  |  |  |  |  |
| **kynurenine** | **4.07E-02** | **7.39** | **0.62** | **2** |  |  |  |  |  | |  |  |  |  |  |
| **PC aa C34:4** | **9.48E-03** | **10.33** | **0.75** | **2** |  |  |  |  |  | |  |  |  |  |  |
| **PC ae C34:1** | **1.66E-02** | **11.47** | **0.55** | **1** |  |  |  |  |  | |  |  |  |  |  |
| **PC aa C36:6** | **8.10E-03** | **11.56** | **0.72** | **2** |  |  |  |  |  | |  |  |  |  |  |
| **PC ae C30:0** | **4.95E-02** | **12.05** | **0.57** | **2** |  |  |  |  |  | |  |  |  |  |  |
| **PC aa C36:0** | **9.38E-03** | **12.06** | **0.44** | **1** |  |  |  |  |  | |  |  |  |  |  |
| **SM C20:2** | **1.99E-02** | **12.10** | **0.53** | **2** |  |  |  |  |  | |  |  |  |  |  |
| **PC ae C34:0** | **4.32E-02** | **12.56** | **0.58** | **3** |  |  |  |  |  | |  |  |  |  |  |
| **AC-C5** | **4.26E-02** | **15.31** | **0.49** | **1** |  |  |  |  |  | |  |  |  |  |  |
| PC aa C42:2 | 3.97E-02 | 22.62 | 0.42 | 2 |  |  |  |  |  | |  |  |  |  |  |
| AC-C14:1 |  |  |  |  |  | 1.30E-03 | 5.19 | 0.57 | 2 | |  | 1.18E-03 | 3.36 | 0.86 | 2 |
| **AC-C18** |  |  |  |  |  | **3.99E-02** | **9.05** | **0.50** | **2** | |  | **3.71E-02** | **9.32** | **0.59** | **1** |
| **PC aa C36:2** |  |  |  |  |  | **8.67E-04** | **19.75** | **0.64** | **3** | |  | **2.88E-02** | **19.73** | **0.65** | **3** |
| **lysoPC a C16:0** |  |  |  |  |  | **2.39E-03** | **20.21** | **0.60** | **1** | |  | **4.69E-02** | **21.32** | **0.61** | **1** |
| **lysoPC a C18:0** |  |  |  |  |  | **6.46E-04** | **22.02** | **0.67** | **1** | |  | 4.77E-03 | 23.18 | 0.84 | 2 |
| **lysoPC a C26:0** |  |  |  |  |  | **1.83E-02** | **10.78** | **0.49** | **1** | |  |  |  |  |  |
| **ornithine** |  |  |  |  |  | **1.11E-02** | **14.59** | **0.50** | **1** | |  |  |  |  |  |
| **AC-C0** |  |  |  |  |  | **8.88E-03** | **15.61** | **0.49** | **2** | |  |  |  |  |  |
| **PC aa C36:4** |  |  |  |  |  | **1.43E-02** | **15.67** | **0.46** | **1** | |  |  |  |  |  |
| **PC ae C44:3** |  |  |  |  |  | **2.82E-02** | **15.97** | **0.25** | **1** | |  |  |  |  |  |
| **PC ae C40:1** |  |  |  |  |  | **2.46E-02** | **20.45** | **0.40** | **1** | |  |  |  |  |  |
| **lysoPC a C16:1** |  |  |  |  |  | **2.43E-02** | **20.69** | **0.46** | **0** | |  |  |  |  |  |
| **lysoPC a C17:0** |  |  |  |  |  | **3.21E-03** | **20.77** | **0.62** | **0** | |  |  |  |  |  |
| **threonine** |  |  |  |  |  | **4.03E-02** | **21.09** | **0.35** | **3** | |  |  |  |  |  |
| **taurine** |  |  |  |  |  |  |  |  |  | |  | **1.37E-02** | **11.32** | **0.63** | **2** |

Cosinor analysis was performed on the mean z-score values for all subjects. Peak time (acrophase), amplitude and significance of a cosine fit (p < 0.05) were determined, n is the number of participants from the study group that displayed significant individual cosine rhythms. Metabolites in bold represent those that peak during lights on (06:30 – 22.30 h).
