# Supplementary material for: Twenty-four-hour rhythmicity of circulating metabolites: effect of body mass and type 2 diabetes
Source: FASEB J. 2017 Aug 18;31(12):5557–67. doi: 10.1096/fj.201700323R (PMC5690388; doi:10.1096/fj.201700323R)
Supplement: Supplemental Data [file supp_fj.201700323R_Supplemental_Table2.docx]

**Table S2** OPLS-DA loadings (p[1]) of the metabolites in models

comparing lean vs OW/OB and OW/OB vs T2DM groups

| Lean vs OW/OB p[1] | | OW/OB vs T2DM p[1] | |
| --- | --- | --- | --- |
| glutamate | 0.13 | alanine | 0.23 |
| tyrosine | 0.11 | PC aa C36:5 | 0.22 |
| AC-C5 | 0.10 | PC aa C38:4 | 0.22 |
| ornithine | 0.10 | t4-OH-Pro | 0.21 |
| AC-C18:1 | 0.10 | alpha-AAA | 0.21 |
| AC-C4 | 0.09 | PC aa C36:4 | 0.21 |
| kynurenine | 0.09 | sarcosine | 0.19 |
| AC-C18:2 | 0.08 | PC aa C34:4 | 0.17 |
| histdine | 0.08 | AC-C3 | 0.17 |
| AC-C16 | 0.07 | PC aa C32:1 | 0.16 |
| valine | 0.07 | proline | 0.15 |
| sarcosine | 0.07 | PC aa C36:6 | 0.14 |
| isoleucine | 0.07 | tyrosine | 0.13 |
| leucine | 0.06 | glycine | 0.13 |
| ADMA | 0.06 | PC aa C32:0 | 0.13 |
| creatinine | 0.06 | PC aa C38:3 | 0.13 |
| tryptophan | 0.05 | PC aa C40:4 | 0.11 |
| proline | 0.04 | phenylalanine | 0.11 |
| methionine | 0.04 | PC aa C38:5 | 0.11 |
| AC-C3 | 0.04 | PC aa C36:1 | 0.11 |
| AC-C0 | 0.03 | PC ae C36:0 | 0.10 |
| AC-C2 | 0.03 | SM C26:1 | 0.10 |
| alpha-AAA | 0.03 | PC aa C42:5 | 0.09 |
| lysine | 0.03 | methionine | 0.09 |
| threonine | 0.03 | PC aa C40:3 | 0.09 |
| phenylalanine | 0.03 | lysine | 0.09 |
| PC aa C40:6 | 0.02 | PC aa C34:1 | 0.09 |
| SM C20:2 | 0.02 | lysoPC a C26:1 | 0.08 |
| PC aa C38:6 | 0.01 | kynurenine | 0.08 |
| t4-OH-Pro | 0.01 | PC aa C40:6 | 0.07 |
| AC-C18 | 0.01 | isoleucine | 0.07 |
| alanine | 0.01 | SM C20:2 | 0.07 |
| PC aa C36:4 | 0.00 | valine | 0.07 |
| PC aa C28:1 | 0.00 | tryptophan | 0.06 |
| serine | 0.00 | PC aa C40:5 | 0.06 |
| PC aa C38:4 | 0.00 | AC-C4 | 0.06 |
| PC ae C30:2 | 0.00 | PC aa C36:0 | 0.06 |
| SM (OH) C14:1 | -0.01 | asparagine | 0.06 |
| PC aa C34:2 | -0.01 | PC aa C34:3 | 0.06 |
| PC aa C32:3 | -0.02 | PC aa C36:3 | 0.05 |
| PC aa C32:1 | -0.02 | PC aa C32:3 | 0.05 |
| PC aa C38:0 | -0.02 | lysoPC a C20:4 | 0.05 |
| taurine | -0.02 | PC ae C32:1 | 0.05 |
| PC ae C38:4 | -0.02 | PC ae C34:0 | 0.04 |
| PC aa C34:4 | -0.02 | PC ae C30:2 | 0.04 |
| SDMA | -0.02 | AC-C0 | 0.04 |
| SM C16:1 | -0.02 | SM (OH) C24:1 | 0.04 |
| PC ae C40:6 | -0.03 | PC aa C42:1 | 0.04 |
| AC-C14:1 | -0.03 | PC aa C38:6 | 0.04 |
| PC ae C36:4 | -0.03 | taurine | 0.03 |
| lysoPC a C26:1 | -0.03 | SM C26:0 | 0.03 |
| SM (OH) C16:1 | -0.03 | PC aa C36:2 | 0.03 |
| serotonin | -0.03 | lysoPC a C24:0 | 0.02 |
| SM C18:0 | -0.03 | leucine | 0.02 |
| glycine | -0.04 | PC ae C38:4 | 0.02 |
| PC ae C38:6 | -0.04 | lysoPC a C20:3 | 0.02 |
| PC aa C36:0 | -0.04 | SM (OH) C22:1 | 0.02 |
| lysoPC a C24:0 | -0.04 | AC-C2 | 0.02 |
| PC ae C40:2 | -0.04 | SM C24:1 | 0.02 |
| PC ae C34:0 | -0.04 | PC ae C40:3 | 0.02 |
| PC ae C38:5 | -0.04 | PC ae C42:1 | 0.02 |
| lysoPC a C16:1 | -0.04 | PC ae C34:1 | 0.01 |
| citrulline | -0.04 | PC ae C30:0 | 0.01 |
| SM C18:1 | -0.05 | PC ae C44:6 | 0.01 |
| arginine | -0.05 | PC ae C36:1 | 0.01 |
| lysoPC a C28:1 | -0.05 | PC aa C42:4 | 0.01 |
| PC ae C44:5 | -0.05 | SM C18:0 | 0.01 |
| lysoPC a C26:0 | -0.05 | PC ae C44:3 | 0.01 |
| lysoPC a C16:0 | -0.06 | PC ae C36:4 | 0.01 |
| PC aa C34:1 | -0.06 | PC ae C38:3 | 0.01 |
| lysoPC a C20:4 | -0.06 | PC aa C34:2 | 0.01 |
| PC aa C32:0 | -0.06 | SM C24:0 | 0.00 |
| PC ae C34:1 | -0.07 | PC aa C28:1 | 0.00 |
| PC aa C36:6 | -0.07 | PC ae C42:2 | 0.00 |
| PC aa C36:2 | -0.07 | lysoPC a C26:0 | 0.00 |
| SM (OH) C24:1 | -0.07 | PC ae C40:2 | 0.00 |
| PC ae C36:1 | -0.08 | arginine | 0.00 |
| PC aa C38:3 | -0.08 | PC ae C40:4 | -0.01 |
| PC ae C38:0 | -0.08 | PC ae C38:0 | -0.01 |
| PC ae C42:5 | -0.08 | PC ae C40:1 | -0.01 |
| glycine | -0.08 | AC-C16 | -0.01 |
| PC ae C36:5 | -0.09 | glycine | -0.01 |
| PC aa C34:3 | -0.09 | PC aa C42:2 | -0.01 |
| SM (OH) C22:1 | -0.09 | PC ae C42:3 | -0.01 |
| SM C24:1 | -0.09 | SM (OH) C22:2 | -0.01 |
| PC ae C36:2 | -0.09 | PC ae C34:3 | -0.02 |
| SM C24:0 | -0.09 | lysoPC a C16:1 | -0.02 |
| SM C16:0 | -0.09 | PC ae C36:5 | -0.02 |
| SM C26:1 | -0.09 | PC ae C36:3 | -0.02 |
| PC aa C42:1 | -0.09 | SM C18:1 | -0.02 |
| lysoPC a C17:0 | -0.09 | PC ae C38:5 | -0.03 |
| PC ae C32:2 | -0.09 | serotonin | -0.03 |
| PC aa C42:2 | -0.10 | AC-C14:1 | -0.03 |
| PC aa C42:0 | -0.10 | PC aa C38:0 | -0.03 |
| PC aa C36:3 | -0.10 | SM C16:1 | -0.03 |
| PC ae C38:3 | -0.10 | threonine | -0.04 |
| PC aa C40:2 | -0.10 | SM (OH) C14:1 | -0.04 |
| lysoPC a C28:0 | -0.10 | PC ae C32:2 | -0.04 |
| PC aa C42:4 | -0.10 | PC ae C42:4 | -0.05 |
| asparagine | -0.10 | PC ae C34:2 | -0.05 |
| PC ae C40:5 | -0.10 | lysoPC a C28:1 | -0.05 |
| SM (OH) C22:2 | -0.11 | serine | -0.06 |
| PC ae C40:4 | -0.11 | SDMA | -0.06 |
| PC ae C44:6 | -0.11 | lysoPC a C28:0 | -0.06 |
| lysoPC a C18:0 | -0.11 | PC ae C38:6 | -0.06 |
| PC ae C32:1 | -0.11 | citrulline | -0.06 |
| PC ae C30:0 | -0.12 | PC ae C40:5 | -0.06 |
| SM C26:0 | -0.12 | SM (OH) C16:1 | -0.07 |
| PC aa C36:5 | -0.12 | PC aa C42:0 | -0.07 |
| PC aa C36:1 | -0.12 | creatinine | -0.07 |
| PC aa C42:5 | -0.12 | AC-C5 | -0.07 |
| PC ae C44:4 | -0.12 | lysoPC a C18:0 | -0.08 |
| PC aa C38:5 | -0.12 | PC ae C40:6 | -0.08 |
| PC ae C40:1 | -0.12 | ornithine | -0.08 |
| PC ae C36:0 | -0.12 | lysoPC a C16:0 | -0.08 |
| PC ae C42:3 | -0.13 | PC ae C36:2 | -0.08 |
| PC aa C40:5 | -0.13 | PC ae C42:5 | -0.09 |
| PC ae C34:2 | -0.14 | SM C16:0 | -0.10 |
| PC ae C42:4 | -0.14 | ADMA | -0.10 |
| lysoPC a C18:1 | -0.14 | PC ae C44:5 | -0.10 |
| PC ae C44:3 | -0.15 | AC-C18:2 | -0.10 |
| lysoPC a C18:2 | -0.15 | AC-C18 | -0.10 |
| PC ae C42:1 | -0.16 | AC-C18:1 | -0.10 |
| lysoPC a C20:3 | -0.17 | lysoPC a C18:1 | -0.10 |
| PC aa C40:4 | -0.17 | lysoPC a C18:2 | -0.11 |
| PC ae C36:3 | -0.17 | PC ae C44:4 | -0.12 |
| PC ae C40:3 | -0.17 | glutamate | -0.13 |
| PC ae C42:2 | -0.19 | PC aa C40:2 | -0.16 |
| PC aa C40:3 | -0.20 | lysoPC a C17:0 | -0.17 |
| PC ae C34:3 | -0.22 | histidine | -0.22 |
