# Supplementary material for: Twenty-four-hour rhythmicity of circulating metabolites: effect of body mass and type 2 diabetes
Source: FASEB J. 2017 Aug 18;31(12):5557–67. doi: 10.1096/fj.201700323R (PMC5690388; doi:10.1096/fj.201700323R)
Supplement: Supplemental Data [file supp_fj.201700323R_Supplemental_Table3.docx]

**Table S3.** Metabolite concentrations (µM, mean ± SEM) and differences between the lean vs OW/OB and OW/OB vs T2DM groups

|  | Concentration (µM) mean ± SEM | | | | | | | | | Lean vs OW/OB | | | | OW/OB vs T2DM | | | |
| --- | --- | --- | --- | --- | --- | --- | --- | --- | --- | --- | --- | --- | --- | --- | --- | --- | --- |
| metabolite | Lean | | | OW/OB | | | T2DM | | | % | FDR | | time max | % | FDR | | time max diff (h) |
|  | (n = 8) | | | (n = 9) | | | (n = 6) | | | change | group | time | diff (h) | change | group | time |  |
| alanine | 367.9 | ± | 21.5 | 376.8 | ± | 17.4 | 470.7 | ± | 23.8 | 2.4 | 5.95E-01 | **2.76E-11** | 01:00 | 24.9 | **5.11E-10** | **3.24E-07** | 7:00 |
| arginine | 105.5 | ± | 5.36 | 99.1 | ± | 4.87 | 97.6 | ± | 7.89 | -6.1 | **2.99E-03** | **8.85E-06** | 05:00 | -1.5 | 7.52E-01 | **1.57E-04** | 23:00 |
| asparagine | 63.8 | ± | 5.9 | 55.3 | ± | 2.03 | 57.9 | ± | 2.51 | -13.3 | **1.96E-06** | 1.54E-01 | 21:00 | 4.6 | 2.88E-01 | 5.99E-02 | 1:00 |
| citrulline | 45.4 | ± | 4.69 | 41.9 | ± | 1.98 | 39.2 | ± | 5.25 | -7.7 | **2.75E-02** | **8.49E-08** | 05:00 | -6.4 | 2.39E-01 | **1.33E-03** | 1:00 |
| glutamine | 720.5 | ± | 22.7 | 698.6 | ± | 20.4 | 650.6 | ± | 23.4 | -3 | 1.02E-01 | **9.16E-03** | 05:00 | -6.9 | **3.80E-04** | **1.77E-03** | 23:00 |
| glutamate | 53.1 | ± | 3.73 | 73 | ± | 7.01 | 88.8 | ± | 10.7 | 37.4 | **2.87E-09** | 2.32E-01 | 01:00 | 21.6 | **3.05E-04** | 6.38E-01 | 9:00 |
| glycine | 223.4 | ± | 16.3 | 202.1 | ± | 10.6 | 200.4 | ± | 16.4 | -9.6 | **7.89E-10** | **3.09E-06** | 05:00 | -0.9 | 8.63E-01 | **6.23E-04** | 9:00 |
| histidine | 96.5 | ± | 2.6 | 102.1 | ± | 5.07 | 86.7 | ± | 3.59 | 5.7 | **3.19E-03** | 7.18E-02 | 13:00 | -15.1 | **1.59E-13** | **2.04E-03** | 23:00 |
| isoleucine | 93.1 | ± | 3.35 | 99.7 | ± | 4.82 | 103.5 | ± | 3.11 | 7.1 | **1.99E-02** | **7.59E-08** | 07:00 | 3.8 | 3.05E-01 | **2.70E-11** | 15:00 |
| leucine | 158.4 | ± | 4.02 | 168.2 | ± | 9.15 | 169.6 | ± | 7.65 | 6.2 | 5.27E-02 | **2.71E-04** | 19:00 | 0.8 | 9.39E-01 | **1.42E-08** | 15:00 |
| lysine | 292.3 | ± | 9.02 | 298.6 | ± | 14.8 | 313.3 | ± | 13 | 2.1 | 4.77E-01 | **5.35E-10** | 05:00 | 4.9 | 8.48E-02 | **4.63E-09** | 5:00 |
| methionine | 33.6 | ± | 0.89 | 34.6 | ± | 0.78 | 36.4 | ± | 1.8 | 3.1 | 2.93E-01 | **2.67E-15** | 19:00 | 5.2 | 8.18E-02 | **2.88E-14** | 7:00 |
| ornithine | 67.8 | ± | 4.05 | 77 | ± | 7.23 | 70.1 | ± | 5.35 | 13.6 | **1.07E-06** | **1.49E-02** | 19:00 | -8.9 | **3.24E-03** | **2.56E-04** | 15:00 |
| phenylalanine | 90.9 | ± | 4.14 | 92.7 | ± | 2.92 | 97.9 | ± | 4.57 | 2 | 4.12E-01 | **6.11E-11** | 01:00 | 5.6 | **1.40E-02** | **2.36E-10** | 7:00 |
| proline | 310.6 | ± | 16.8 | 326.9 | ± | 12.9 | 359.4 | ± | 20.3 | 5.2 | **2.82E-02** | **1.33E-24** | 13:00 | 9.9 | **5.90E-05** | **3.51E-19** | 5:00 |
| serine | 119.8 | ± | 4.21 | 118.3 | ± | 6.28 | 111.8 | ± | 5.8 | -1.3 | 6.70E-01 | **2.61E-03** | 05:00 | -5.5 | **1.64E-02** | **4.54E-05** | 15:00 |
| threonine | 136.4 | ± | 5.72 | 142.4 | ± | 5.88 | 138.6 | ± | 8.22 | 4.4 | 2.45E-01 | 1.94E-01 | 01:00 | -2.7 | 6.35E-01 | 1.30E-01 | 1:00 |
| tryptophan | 74.6 | ± | 3.49 | 79.9 | ± | 5.38 | 83.7 | ± | 6.08 | 7.1 | **2.41E-03** | **2.28E-07** | 01:00 | 4.8 | 1.10E-01 | **1.67E-06** | 11:00 |
| tyrosine | 87.5 | ± | 3.51 | 99.1 | ± | 4.92 | 110.9 | ± | 9.84 | 13.2 | **3.98E-08** | **1.12E-15** | 19:00 | 11.9 | **6.19E-06** | **1.14E-16** | 7:00 |
| valine | 207.9 | ± | 5.86 | 219.1 | ± | 7.06 | 225.4 | ± | 5.51 | 5.4 | **2.54E-02** | 1.02E-01 | 09:00 | 2.9 | 3.47E-01 | 1.64E-01 | 5:00 |
| ADMA | 0.563 | ± | 0.03 | 0.614 | ± | 0.03 | 0.568 | ± | 0.03 | 9.1 | **1.01E-02** | 3.22E-01 | 01:00 | -7.5 | 6.26E-02 | 1.91E-01 | 1:00 |
| alpha-AAA | 1.21 | ± | 0.1 | 1.25 | ± | 0.09 | 1.5 | ± | 0.11 | 3.6 | 1.51E-01 | **2.10E-09** | 09:00 | 19.9 | **1.58E-16** | **3.49E-11** | 21:00 |
| creatinine | 69 | ± | 3.19 | 76.9 | ± | 4.28 | 72.9 | ± | 6.65 | 11.3 | **8.49E-03** | 2.36E-01 | 01:00 | -5.2 | 3.66E-01 | 4.08E-01 | 1:00 |
| kynurenine | 1.86 | ± | 0.16 | 2.16 | ± | 0.21 | 2.29 | ± | 0.12 | 16.2 | **1.56E-08** | **3.17E-06** | 01:00 | 5.6 | 9.57E-02 | **2.04E-03** | 11:00 |
| sarcosine | 1.57 | ± | 0.14 | 1.79 | ± | 0.09 | 2.16 | ± | 0.1 | 13.7 | **6.71E-04** | **1.91E-06** | 01:00 | 20.6 | **3.45E-07** | **7.25E-09** | 11:00 |
| SDMA | 0.542 | ± | 0.03 | 0.53 | ± | 0.02 | 0.509 | ± | 0.03 | -2.2 | 6.51E-01 | 8.50E-02 | 01:00 | -3.9 | 3.95E-01 | 4.11E-01 | 1:00 |
| serotonin | 0.209 | ± | 0.02 | 0.179 | ± | 0.01 | 0.165 | ± | 0.03 | -14.2 | 3.79E-01 | 4.80E-01 | 17:00 | -7.8 | 7.67E-01 | 2.02E-01 | 9:00 |
| t4-OH-Pro | 4.91 | ± | 0.19 | 4.96 | ± | 0.15 | 5.82 | ± | 0.6 | 1 | 7.35E-01 | 2.47E-01 | 01:00 | 17.5 | **6.28E-16** | **1.23E-02** | 11:00 |
| taurine | 61.7 | ± | 3.66 | 59.7 | ± | 3.23 | 61.9 | ± | 4.63 | -3.2 | 7.40E-01 | 7.32E-01 | 11:00 | 3.6 | 8.13E-01 | 4.32E-01 | 1:00 |
| AC-C0 | 41 | ± | 2.91 | 43.3 | ± | 4.81 | 46.3 | ± | 4.38 | 5.5 | **9.50E-03** | **1.17E-02** | 13:00 | 7 | **2.45E-03** | **2.34E-02** | 17:00 |
| AC-C2 | 4.828 | ± | 0.29 | 5.14 | ± | 0.42 | 5.254 | ± | 0.42 | 6.4 | **4.56E-02** | **4.74E-33** | 19:00 | 2.2 | 6.30E-01 | **2.16E-23** | 17:00 |
| AC-C3 | 0.266 | ± | 0.02 | 0.283 | ± | 0.03 | 0.339 | ± | 0.03 | 6.4 | **2.05E-02** | **3.18E-20** | 01:00 | 19.7 | **5.28E-08** | **5.14E-14** | 11:00 |
| AC-C4 | 0.173 | ± | 0.01 | 0.196 | ± | 0.01 | 0.204 | ± | 0.02 | 12.8 | **9.12E-07** | **2.62E-10** | 19:00 | 4.4 | 1.68E-01 | **3.97E-12** | 17:00 |
| AC-C5 | 0.148 | ± | 0.01 | 0.173 | ± | 0.01 | 0.161 | ± | 0.01 | 16.6 | **1.91E-06** | **2.57E-06** | 19:00 | -6.8 | 5.59E-02 | **2.35E-06** | 23:00 |
| AC-C14:1 | 0.061 | ± | 0 | 0.058 | ± | 0 | 0.056 | ± | 0.01 | -4.3 | 2.47E-01 | **7.56E-03** | 15:00 | -3.3 | 4.70E-01 | **1.21E-05** | 11:00 |
| AC-C16 | 0.071 | ± | 0.01 | 0.082 | ± | 0.01 | 0.08 | ± | 0.01 | 15.3 | **6.41E-06** | **6.27E-18** | 23:00 | -2.2 | 6.58E-01 | **1.33E-17** | 23:00 |
| AC-C18 | 0.028 | ± | 0 | 0.028 | ± | 0 | 0.024 | ± | 0 | 1.3 | 8.39E-01 | **3.85E-03** | 17:00 | -13.7 | **1.73E-03** | **5.50E-04** | 23:00 |
| AC-C18:1 | 0.102 | ± | 0.01 | 0.122 | ± | 0.01 | 0.107 | ± | 0.01 | 19.1 | **5.99E-07** | **2.57E-06** | 09:00 | -11.8 | **5.80E-04** | **2.36E-05** | 23:00 |
| AC-C18:2 | 0.033 | ± | 0 | 0.037 | ± | 0 | 0.032 | ± | 0 | 13.5 | **1.52E-04** | **4.52E-02** | 09:00 | -12.8 | **2.90E-04** | 1.67E-01 | 23:00 |
| lysoPC a C16:0 | 72.7 | ± | 2.54 | 67.9 | ± | 4.98 | 62.5 | ± | 10.45 | -6.7 | **4.49E-03** | **3.05E-05** | 09:00 | -7.9 | **5.53E-03** | **1.31E-05** | 7:00 |
| lysoPC a C16:1 | 2.47 | ± | 0.08 | 2.32 | ± | 0.27 | 2.3 | ± | 0.31 | -6.2 | **1.49E-02** | **2.64E-03** | 09:00 | -0.5 | 9.89E-01 | **2.51E-03** | 11:00 |
| lysoPC a C17:0 | 1.3 | ± | 0.06 | 1.11 | ± | 0.12 | 0.88 | ± | 0.14 | -14.2 | **9.59E-10** | **8.21E-05** | 09:00 | -20.5 | **2.30E-13** | **7.00E-06** | 15:00 |
| lysoPC a C18:0 | 16.5 | ± | 0.6 | 14.1 | ± | 1.11 | 12.9 | ± | 2.84 | -14.5 | **9.87E-10** | **2.02E-05** | 09:00 | -8.8 | **1.80E-03** | **1.33E-06** | 7:00 |
| lysoPC a C18:1 | 17.3 | ± | 0.65 | 14.1 | ± | 1 | 12.6 | ± | 1.49 | -18.4 | **5.00E-15** | **1.56E-15** | 09:00 | -10.2 | **2.61E-04** | **3.01E-18** | 15:00 |
| lysoPC a C18:2 | 32.4 | ± | 1.29 | 24.7 | ± | 1.59 | 21.4 | ± | 2.44 | -24 | **1.54E-21** | **1.54E-33** | 09:00 | -13.2 | **6.23E-06** | **2.74E-33** | 15:00 |
| lysoPC a C20:3 | 1.89 | ± | 0.15 | 1.37 | ± | 0.13 | 1.46 | ± | 0.28 | -27.4 | **1.10E-22** | **6.05E-12** | 09:00 | 5.9 | 1.28E-01 | **7.51E-15** | 9:00 |
| lysoPC a C20:4 | 4.84 | ± | 0.16 | 4.37 | ± | 0.45 | 4.64 | ± | 0.56 | -9.6 | **1.44E-04** | **3.92E-15** | 09:00 | 6.1 | 6.48E-02 | **2.02E-18** | 9:00 |
| lysoPC a C24:0 | 0.09 | ± | 0 | 0.085 | ± | 0 | 0.086 | ± | 0 | -5.9 | 2.35E-01 | 4.35E-01 | 05:00 | 1.1 | 9.68E-01 | 3.66E-01 | 11:00 |
| lysoPC a C26:0 | 0.157 | ± | 0.01 | 0.143 | ± | 0.01 | 0.141 | ± | 0.01 | -9.1 | 5.72E-02 | **5.57E-03** | 15:00 | -1.1 | 9.79E-01 | **1.18E-03** | 17:00 |
| lysoPC a C26:1 | 0.07 | ± | 0 | 0.066 | ± | 0 | 0.07 | ± | 0 | -6.2 | 2.29E-01 | 4.64E-01 | 05:00 | 6.3 | 3.11E-01 | **3.90E-02** | 15:00 |
| lysoPC a C28:0 | 0.125 | ± | 0.01 | 0.104 | ± | 0.01 | 0.095 | ± | 0.01 | -16.4 | **2.10E-05** | 3.50E-01 | 15:00 | -9.1 | 1.11E-01 | 7.94E-02 | 17:00 |
| lysoPC a C28:1 | 0.262 | ± | 0.02 | 0.241 | ± | 0.03 | 0.227 | ± | 0.02 | -8 | **2.99E-03** | 5.83E-02 | 11:00 | -5.8 | 6.25E-02 | **2.48E-02** | 11:00 |
| PC aa C28:1 | 1.78 | ± | 0.08 | 1.77 | ± | 0.15 | 1.76 | ± | 0.24 | -0.6 | 8.54E-01 | 7.00E-01 | 07:00 | -0.5 | 9.89E-01 | 1.43E-01 | 23:00 |
| PC aa C32:0 | 11.7 | ± | 0.66 | 10.9 | ± | 0.74 | 12.7 | ± | 1.81 | -6.3 | **3.84E-03** | 1.59E-01 | 05:00 | 16.1 | **5.11E-10** | 6.72E-02 | 17:00 |
| PC aa C32:1 | 10.3 | ± | 1.09 | 10.3 | ± | 2.64 | 15.4 | ± | 3.32 | -0.4 | 9.19E-01 | **1.34E-17** | 01:00 | 50.1 | **3.36E-22** | **2.28E-09** | 17:00 |
| PC aa C32:3 | 0.323 | ± | 0.01 | 0.321 | ± | 0.04 | 0.341 | ± | 0.05 | -0.6 | 8.34E-01 | 2.35E-01 | 01:00 | 6.5 | **1.71E-02** | 3.17E-01 | 21:00 |
| PC aa C34:1 | 258.7 | ± | 13.1 | 241.6 | ± | 21.6 | 269.4 | ± | 39.7 | -6.6 | **3.85E-03** | 1.82E-01 | 05:00 | 11.5 | **4.25E-06** | **1.07E-02** | 17:00 |
| PC aa C34:2 | 289.3 | ± | 11.5 | 285 | ± | 15.5 | 286.5 | ± | 31.3 | -1.5 | 7.51E-01 | 6.55E-01 | 15:00 | 0.5 | 9.89E-01 | 9.00E-02 | 23:00 |
| PC aa C34:3 | 16.3 | ± | 0.99 | 14.3 | ± | 1.64 | 15.6 | ± | 2.9 | -12.4 | **1.09E-09** | 3.74E-01 | 09:00 | 9.3 | **9.46E-04** | 3.56E-01 | 17:00 |
| PC aa C34:4 | 1.08 | ± | 0.09 | 1.05 | ± | 0.17 | 1.37 | ± | 0.19 | -3.4 | 1.83E-01 | **2.10E-03** | 09:00 | 31.1 | **3.36E-22** | 2.14E-01 | 17:00 |
| PC aa C36:0 | 4.1 | ± | 0.6 | 3.81 | ± | 0.42 | 4.04 | ± | 0.47 | -7 | **2.12E-02** | **4.69E-02** | 05:00 | 6.1 | 9.75E-02 | 2.46E-01 | 23:00 |
| PC aa C36:1 | 58.5 | ± | 3.94 | 49.6 | ± | 4.55 | 58.4 | ± | 10.9 | -15.3 | **2.74E-14** | **9.93E-03** | 07:00 | 17.7 | **5.11E-10** | **3.23E-03** | 17:00 |
| PC aa C36:2 | 202.8 | ± | 11.9 | 185.1 | ± | 13.8 | 193.5 | ± | 35.6 | -8.7 | **3.27E-05** | **3.35E-03** | 05:00 | 4.5 | 1.06E-01 | **6.09E-06** | 17:00 |
| PC aa C36:3 | 150 | ± | 9.45 | 132.6 | ± | 9.75 | 143.8 | ± | 28 | -11.6 | **2.86E-09** | **1.16E-02** | 05:00 | 8.5 | **1.15E-03** | **4.04E-04** | 17:00 |
| PC aa C36:4 | 162.8 | ± | 5.31 | 163.1 | ± | 14.88 | 201.2 | ± | 18 | 0.2 | 9.23E-01 | 8.07E-02 | 05:00 | 23.4 | **4.26E-18** | **1.88E-04** | 17:00 |
| PC aa C36:5 | 29.2 | ± | 6.55 | 20.9 | ± | 3.45 | 29.1 | ± | 3.16 | -28.4 | **6.98E-34** | **2.66E-05** | 09:00 | 39.3 | **1.28E-25** | **5.46E-06** | 13:00 |
| PC aa C36:6 | 0.604 | ± | 0.09 | 0.52 | ± | 0.08 | 0.632 | ± | 0.07 | -13.8 | **4.52E-09** | **1.66E-03** | 11:00 | 21.6 | **5.34E-12** | **1.40E-02** | 11:00 |
| PC aa C38:0 | 3.316 | ± | 0.43 | 3.228 | ± | 0.32 | 3.1 | ± | 0.28 | -2.6 | 3.38E-01 | 6.42E-01 | 05:00 | -4 | 1.74E-01 | 1.85E-01 | 23:00 |
| PC aa C38:3 | 51.8 | ± | 3.63 | 46.3 | ± | 4.12 | 56.3 | ± | 12.1 | -10.5 | **5.80E-08** | 1.99E-01 | 11:00 | 21.6 | **5.34E-15** | **3.20E-02** | 17:00 |
| PC aa C38:4 | 79.7 | ± | 3.26 | 78.5 | ± | 7.92 | 99.9 | ± | 11 | -1.5 | 6.12E-01 | 5.25E-01 | 05:00 | 27.4 | **7.13E-22** | **4.86E-02** | 17:00 |
| PC aa C38:5 | 61.6 | ± | 4.24 | 52 | ± | 5.13 | 59.3 | ± | 7.16 | -15.6 | **2.78E-16** | 2.54E-01 | 05:00 | 14 | **4.75E-08** | **4.97E-02** | 17:00 |
| PC aa C38:6 | 72.8 | ± | 8.18 | 74.6 | ± | 7.14 | 77.2 | ± | 9.28 | 2.5 | 3.79E-01 | 1.62E-01 | 01:00 | 3.5 | 2.49E-01 | **2.14E-02** | 21:00 |
| PC aa C40:2 | 0.197 | ± | 0.02 | 0.162 | ± | 0.01 | 0.13 | ± | 0.01 | -17.9 | **7.75E-06** | **1.76E-03** | 05:00 | -19.6 | **3.06E-04** | 1.44E-01 | 13:00 |
| PC aa C40:3 | 0.72 | ± | 0.03 | 0.587 | ± | 0.03 | 0.653 | ± | 0.11 | -18.4 | **2.37E-16** | 7.59E-01 | 17:00 | 11.1 | **1.16E-04** | 4.76E-01 | 17:00 |
| PC aa C40:4 | 2.74 | ± | 0.17 | 2.16 | ± | 0.18 | 2.67 | ± | 0.71 | -21.3 | **1.38E-24** | 5.21E-01 | 09:00 | 23.6 | **4.89E-16** | 3.39E-01 | 17:00 |
| PC aa C40:5 | 11.9 | ± | 0.71 | 10 | ± | 0.99 | 10.9 | ± | 2.16 | -16.7 | **2.37E-16** | 2.94E-01 | 05:00 | 9.8 | **2.61E-04** | 2.78E-01 | 17:00 |
| PC aa C40:6 | 23.1 | ± | 2.47 | 24.1 | ± | 3.01 | 26.1 | ± | 2.27 | 4.4 | 6.91E-02 | 5.82E-01 | 01:00 | 8.2 | **1.17E-03** | 2.98E-01 | 17:00 |
| PC aa C42:0 | 0.762 | ± | 0.12 | 0.632 | ± | 0.05 | 0.593 | ± | 0.06 | -17 | **1.42E-16** | 1.83E-01 | 05:00 | -6.2 | **1.32E-02** | 1.27E-01 | 23:00 |
| PC aa C42:1 | 0.379 | ± | 0.05 | 0.322 | ± | 0.02 | 0.333 | ± | 0.03 | -14.9 | **6.05E-12** | 7.59E-01 | 15:00 | 3.3 | 3.09E-01 | 2.13E-01 | 11:00 |
| PC aa C42:2 | 0.235 | ± | 0.03 | 0.202 | ± | 0.02 | 0.2 | ± | 0.02 | -14 | **2.77E-08** | 4.68E-01 | 05:00 | -1.4 | 8.53E-01 | 3.80E-01 | 1:00 |
| PC aa C42:4 | 0.156 | ± | 0 | 0.14 | ± | 0.01 | 0.14 | ± | 0.01 | -10.1 | **6.75E-05** | 8.84E-01 | 05:00 | 0.3 | 9.89E-01 | 9.73E-01 | 3:00 |
| PC aa C42:5 | 0.343 | ± | 0.02 | 0.29 | ± | 0.03 | 0.32 | ± | 0.03 | -15.2 | **1.64E-13** | 8.25E-01 | 05:00 | 10.1 | **2.61E-04** | 2.64E-01 | 17:00 |
| PC ae C30:0 | 0.252 | ± | 0.01 | 0.212 | ± | 0.03 | 0.216 | ± | 0.04 | -15.7 | **6.50E-11** | **1.12E-04** | 07:00 | 1.9 | 6.82E-01 | **1.74E-03** | 5:00 |
| PC ae C30:2 | 0.075 | ± | 0 | 0.074 | ± | 0.01 | 0.079 | ± | 0.01 | -0.5 | 8.92E-01 | 3.32E-01 | 01:00 | 6.1 | 8.54E-02 | 1.43E-01 | 7:00 |
| PC ae C32:1 | 1.99 | ± | 0.16 | 1.68 | ± | 0.17 | 1.81 | ± | 0.37 | -15.4 | **1.52E-11** | 1.90E-01 | 05:00 | 7.7 | **9.27E-03** | 5.99E-02 | 11:00 |
| PC ae C32:2 | 0.662 | ± | 0.05 | 0.588 | ± | 0.05 | 0.569 | ± | 0.06 | -11.1 | **9.70E-08** | 3.53E-01 | 05:00 | -3.3 | 3.09E-01 | **3.75E-02** | 7:00 |
| PC ae C34:0 | 1.93 | ± | 0.07 | 1.82 | ± | 0.25 | 1.95 | ± | 0.32 | -5.6 | **2.84E-02** | **9.58E-05** | 05:00 | 7.1 | **2.09E-02** | **7.14E-04** | 17:00 |
| PC ae C34:1 | 9.21 | ± | 0.38 | 8.43 | ± | 1.02 | 8.6 | ± | 1.2 | -8.5 | **8.88E-05** | **2.66E-02** | 05:00 | 2 | 6.38E-01 | **1.18E-02** | 17:00 |
| PC ae C34:2 | 10.6 | ± | 0.57 | 8.81 | ± | 0.78 | 8.37 | ± | 1.49 | -17 | **2.67E-15** | 1.25E-01 | 05:00 | -5 | 8.13E-02 | **6.57E-03** | 23:00 |
| PC ae C34:3 | 5.41 | ± | 0.43 | 3.83 | ± | 0.26 | 3.8 | ± | 0.71 | -29.1 | **1.24E-38** | 1.83E-01 | 05:00 | -0.9 | 8.88E-01 | **1.05E-02** | 23:00 |
| PC ae C36:0 | 1.16 | ± | 0.08 | 0.974 | ± | 0.1 | 1.12 | ± | 0.16 | -16.3 | **1.59E-11** | 1.02E-01 | 17:00 | 14.5 | **1.57E-06** | **1.05E-02** | 17:00 |
| PC ae C36:1 | 16.8 | ± | 0.91 | 15 | ± | 1.71 | 15.3 | ± | 1.97 | -11 | **3.57E-07** | **2.23E-02** | 05:00 | 1.9 | 6.82E-01 | **1.07E-02** | 17:00 |
| PC ae C36:2 | 17.3 | ± | 0.99 | 15.1 | ± | 1.52 | 13.6 | ± | 2.35 | -12.9 | **8.80E-10** | 5.39E-02 | 05:00 | -9.6 | **2.37E-04** | **4.05E-04** | 23:00 |
| PC ae C36:3 | 5.83 | ± | 0.26 | 4.71 | ± | 0.33 | 4.65 | ± | 0.93 | -19.1 | **1.35E-18** | 5.89E-01 | 05:00 | -1.4 | 7.77E-01 | 1.42E-01 | 17:00 |
| PC ae C36:4 | 12.7 | ± | 0.54 | 12.2 | ± | 0.91 | 12.3 | ± | 1.4 | -3.8 | 1.18E-01 | 4.85E-01 | 05:00 | 0.9 | 9.01E-01 | 1.29E-01 | 7:00 |
| PC ae C36:5 | 8.89 | ± | 0.46 | 8.06 | ± | 0.54 | 7.95 | ± | 0.79 | -9.4 | **2.07E-06** | 7.25E-01 | 05:00 | -1.3 | 7.67E-01 | 1.76E-01 | 7:00 |
| PC ae C38:0 | 3.37 | ± | 0.37 | 2.92 | ± | 0.35 | 2.9 | ± | 0.35 | -13.4 | **3.33E-10** | 4.35E-01 | 07:00 | -0.6 | 9.69E-01 | 5.74E-02 | 7:00 |
| PC ae C38:3 | 7.81 | ± | 0.47 | 6.83 | ± | 0.65 | 6.95 | ± | 1.21 | -12.6 | **8.29E-10** | 2.41E-01 | 05:00 | 1.8 | 6.74E-01 | **2.64E-02** | 17:00 |
| PC ae C38:4 | 12 | ± | 0.54 | 11.7 | ± | 1.06 | 12.0 | ± | 1.44 | -2.8 | 2.70E-01 | 3.79E-01 | 05:00 | 2.7 | 3.81E-01 | **4.43E-02** | 17:00 |
| PC ae C38:5 | 11.9 | ± | 0.58 | 11.4 | ± | 0.65 | 11.1 | ± | 0.99 | -3.9 | 9.47E-02 | 7.68E-01 | 05:00 | -2.8 | 3.66E-01 | 3.80E-01 | 23:00 |
| PC ae C38:6 | 6.15 | ± | 0.63 | 5.83 | ± | 0.45 | 5.49 | ± | 0.52 | -5.2 | **1.86E-02** | 5.21E-01 | 05:00 | -5.8 | **2.60E-02** | 2.10E-01 | 7:00 |
| PC ae C40:1 | 1.66 | ± | 0.12 | 1.39 | ± | 0.1 | 1.38 | ± | 0.2 | -16 | **7.28E-12** | 6.66E-02 | 15:00 | -0.7 | 9.78E-01 | **8.74E-04** | 23:00 |
| PC ae C40:2 | 2.33 | ± | 0.2 | 2.21 | ± | 0.25 | 2.2 | ± | 0.18 | -5.4 | **2.44E-02** | 6.42E-01 | 05:00 | -0.3 | 9.89E-01 | 1.59E-01 | 11:00 |
| PC ae C40:3 | 1.8 | ± | 0.05 | 1.5 | ± | 0.1 | 1.54 | ± | 0.18 | -16.8 | **1.86E-16** | 5.61E-01 | 05:00 | 2.6 | 4.26E-01 | 5.27E-02 | 17:00 |
| PC ae C40:4 | 2.21 | ± | 0.11 | 1.96 | ± | 0.11 | 1.96 | ± | 0.25 | -11.5 | **2.77E-08** | 7.40E-01 | 05:00 | 0.1 | 9.89E-01 | 1.94E-01 | 11:00 |
| PC ae C40:5 | 4.51 | ± | 0.19 | 4.04 | ± | 0.26 | 3.86 | ± | 0.41 | -10.4 | **1.42E-07** | 7.51E-01 | 05:00 | -4.6 | 8.48E-02 | 4.70E-01 | 23:00 |
| PC ae C40:6 | 4.7 | ± | 0.42 | 4.51 | ± | 0.39 | 4.15 | ± | 0.4 | -3.9 | 8.95E-02 | 4.89E-01 | 05:00 | -8.1 | **7.04E-04** | 2.07E-01 | 7:00 |
| PC ae C42:1 | 0.45 | ± | 0.02 | 0.367 | ± | 0.02 | 0.374 | ± | 0.05 | -18.4 | **8.36E-15** | 1.75E-01 | 15:00 | 2 | 6.98E-01 | 6.66E-02 | 17:00 |
| PC ae C42:2 | 0.756 | ± | 0.04 | 0.604 | ± | 0.05 | 0.605 | ± | 0.1 | -20.1 | **5.52E-21** | 4.68E-01 | 05:00 | 0.3 | 9.89E-01 | 1.94E-01 | 17:00 |
| PC ae C42:3 | 0.907 | ± | 0.1 | 0.757 | ± | 0.05 | 0.746 | ± | 0.11 | -16.6 | **6.29E-14** | 5.95E-01 | 15:00 | -1.5 | 7.67E-01 | 1.91E-01 | 23:00 |
| PC ae C42:4 | 0.813 | ± | 0.07 | 0.667 | ± | 0.03 | 0.641 | ± | 0.1 | -18 | **3.69E-12** | 8.80E-01 | 05:00 | -3.9 | 2.93E-01 | 5.51E-01 | 23:00 |
| PC ae C42:5 | 1.93 | ± | 0.14 | 1.75 | ± | 0.09 | 1.64 | ± | 0.16 | -9.2 | **1.00E-05** | 3.42E-01 | 05:00 | -6.7 | **5.19E-03** | 1.63E-01 | 7:00 |
| PC ae C44:3 | 0.103 | ± | 0.01 | 0.085 | ± | 0 | 0.086 | ± | 0.01 | -17.4 | **3.41E-08** | 3.78E-01 | 07:00 | 0.5 | 9.89E-01 | 5.51E-01 | 7:00 |
| PC ae C44:4 | 0.414 | ± | 0.03 | 0.357 | ± | 0.02 | 0.325 | ± | 0.04 | -13.7 | **4.28E-09** | 8.80E-01 | 15:00 | -9 | **5.98E-04** | 3.40E-01 | 23:00 |
| PC ae C44:5 | 1.29 | ± | 0.13 | 1.2 | ± | 0.06 | 1.1 | ± | 0.13 | -6.8 | **3.71E-03** | 7.16E-01 | 05:00 | -8.5 | **1.10E-03** | 4.08E-01 | 23:00 |
| PC ae C44:6 | 1.04 | ± | 0.13 | 0.87 | ± | 0.05 | 0.88 | ± | 0.11 | -16.2 | **2.09E-14** | 8.18E-01 | 15:00 | 1.1 | 8.45E-01 | 4.22E-01 | 11:00 |
| SM (OH) C14:1 | 7.51 | ± | 0.35 | 7.43 | ± | 0.52 | 7.09 | ± | 0.99 | -1.1 | 7.06E-01 | 4.64E-01 | 15:00 | -4.5 | 5.99E-02 | 6.08E-02 | 7:00 |
| SM (OH) C16:1 | 4.43 | ± | 0.19 | 4.25 | ± | 0.31 | 3.95 | ± | 0.39 | -4.2 | 7.04E-02 | 7.13E-01 | 05:00 | -7 | **7.18E-03** | 3.34E-01 | 1:00 |
| SM (OH) C22:1 | 32.6 | ± | 1.35 | 29.4 | ± | 2.03 | 29.8 | ± | 3.09 | -10 | **5.87E-06** | 7.63E-01 | 11:00 | 1.4 | 7.85E-01 | 3.39E-01 | 11:00 |
| SM (OH) C22:2 | 41 | ± | 1.71 | 35.8 | ± | 2.78 | 35.0 | ± | 3.45 | -12.8 | **4.00E-09** | 7.59E-01 | 11:00 | -2.2 | 5.51E-01 | 3.47E-01 | 11:00 |
| SM (OH) C24:1 | 1.43 | ± | 0.07 | 1.31 | ± | 0.09 | 1.36 | ± | 0.13 | -8.8 | **4.60E-04** | 7.93E-01 | 11:00 | 3.6 | 3.47E-01 | 4.37E-01 | 11:00 |
| SM C16:0 | 153.9 | ± | 6.99 | 139.6 | ± | 6.37 | 127.6 | ± | 14.6 | -9.3 | **4.73E-06** | 7.39E-01 | 15:00 | -8.6 | **3.05E-04** | 3.38E-01 | 7:00 |
| SM C16:1 | 22.7 | ± | 0.98 | 22.1 | ± | 1.46 | 21.3 | ± | 3.39 | -2.7 | 3.13E-01 | 4.85E-01 | 05:00 | -3.4 | 2.83E-01 | 9.16E-02 | 19:00 |
| SM C18:0 | 31.7 | ± | 0.99 | 30.5 | ± | 1.77 | 30.3 | ± | 1.68 | -3.7 | 1.25E-01 | 4.68E-01 | 11:00 | -0.8 | 9.22E-01 | 4.22E-01 | 11:00 |
| SM C18:1 | 15.1 | ± | 0.48 | 14.3 | ± | 1.01 | 13.9 | ± | 1.38 | -4.8 | 5.51E-02 | 2.89E-01 | 11:00 | -3 | 3.67E-01 | 1.51E-01 | 13:00 |
| SM C20:2 | 0.839 | ± | 0.03 | 0.859 | ± | 0.09 | 0.931 | ± | 0.11 | 2.4 | 4.64E-01 | 3.14E-01 | 23:00 | 8.3 | **2.31E-03** | 1.80E-01 | 11:00 |
| SM C24:0 | 29.4 | ± | 0.56 | 26.8 | ± | 0.89 | 26.7 | ± | 3.61 | -8.7 | **1.44E-04** | 7.63E-01 | 11:00 | -0.3 | 9.89E-01 | 1.42E-01 | 11:00 |
| SM C24:1 | 172.8 | ± | 5.48 | 158.4 | ± | 7.67 | 161.0 | ± | 18.9 | -8.4 | **1.27E-04** | 7.59E-01 | 15:00 | 1.6 | 7.16E-01 | 4.70E-01 | 11:00 |
| SM C26:0 | 0.294 | ± | 0.02 | 0.255 | ± | 0.01 | 0.258 | ± | 0.02 | -13.3 | **4.01E-07** | 9.00E-01 | 11:00 | 1.4 | 8.53E-01 | 9.89E-01 | 11:00 |
| SM C26:1 | 0.476 | ± | 0.05 | 0.41 | ± | 0.03 | 0.442 | ± | 0.03 | -13.9 | **2.91E-07** | 7.40E-01 | 11:00 | 7.9 | **1.88E-02** | 5.33E-01 | 11:00 |

Percentage change in metabolite concentrations between the lean and OW/OB groups and OW/OB and T2DM groups. Higher concentrations are shown as positive values and lower concentrations as negative values. P-values were corrected for multiple comparisons according to the Benjamini-Hochberg False Discovery Rate (FDR) with FDR < 0.05 considered as statistically significant (values in bold). The time of maximum difference (time max diff, h) represents the sampling time point at which the metabolite had the largest %difference between the respective study groups.
